# Sustained delivery of 3D-bioprinted stem cell-derived small extracellular vesicles promotes neural regeneration following traumatic brain injury via the CREB1/PGC-1α/FNDC5 axis

**DOI:** 10.1016/j.bioactmat.2026.10.030

**Published:** 2026-10-31

**Authors:** Ying Ai, Qian Zhang, Tiange Chen, Lin Lin, Ziyang Chen, Ganzhi Liu, Jiacheng Liu, Zhuojing Dong, Yanpeng Jiao, Jingwei Zhang, Yi Zhang, Boxun Liu, Tao Xu, Xin Chen, Jinfang Liu, Liyang Zhang, Yuguo Xia

**Affiliations:** a Department of Neurosurgery, Xiangya Hospital, Central South University, Changsha, Hunan, CN 410008, China; b National Clinical Research Center for Geriatric Disorders, Xiangya Hospital, Central South University, Changsha, Hunan, CN 410008, China; c Research Institute of Brain and Spinal Cord Injury, Xiangya Hospital, Central South University, Changsha, 410008, Hunan, China; d Department of Neurosurgery, Xiangya School of Medicine, Central South University, Changsha, 410008, Hunan, China; e Bio-Intelligent Manufacturing and Living Matter Bioprinting Center, Research Institute of Tsinghua University in Shenzhen, Tsinghua University, Shenzhen, China; f Research and Development Department, Huamei Biotech Co. Ltd., Shenzhen, 518107, China; g Zhongshan School of Medicine, Sun Yat-sen University, Guangzhou, 510080, China; h Department of Neurosurgery, National Regional Center for Neurological Diseases, Xiangya Hospital (Jiangxi), Central South University, Nanchang, Jiangxi, 330038, China; i Hunan Provincial Key Laboratory of Neurocritical Care, China; j Faculty of Medicine, The University of Queensland, Brisbane, Queensland, Australia

**Keywords:** 3D bioprinting, Small extracellular vesicles (sEVs), GelMA hydrogel, Traumatic brain injury, Neural stem cell, Neurogenesis

## Abstract

**Background:**

Clinical translation of stem cell-derived small extracellular vesicles (sEVs) for traumatic brain injury (TBI) is restricted by low yield in 2D culture and rapid *in vivo* clearance.

**Objective:**

To develop an *in situ* sustained delivery system (3D-sEVs@Gel) encapsulating 3D-bioprinted adipose stem cell (ADSC)-derived sEVs (3D-ADSC-sEVs) within GelMA (Gelatin methacryloyl) hydrogel for long-term functional repair after TBI.

**Methods:**

SEVs derived from coaxially bioprinted 3D-ADSCs were isolated, and a GelMA hydrogel-based delivery system was subsequently constructed. Following orthotopic transplantation of the 3D-sEVs@Gel system into the lesion site after TBI, functional recovery, neural stem cell (NSC) proliferation and lineage-associated responses, and the underlying molecular mechanisms were systematically evaluated through behavioral tests, immunofluorescence, multi-omics, and pharmacological inhibition.

**Results:**

SEVs generated using the coaxial alginate-microfiber culture system exhibited an approximately eightfold higher yields and protein loading compared with those obtained from 2D culture. *In vivo*, 3D-sEVs@Gel prolonged local retention, reduced tissue loss, and significantly improved long-term sensorimotor coordination and spatial memory following TBI. In addition, treatment increased the numbers of proliferating cells co-expressing neural progenitor-, neuronal-, and oligodendroglial-lineage-associated markers within the examined neurogenic regions. Mechanistically, 3D-sEV treatment increased nuclear CREB1 levels in NSCs, promoting PGC-1α transcription and subsequent FNDC5 expression, thereby supporting endogenous neurogenesis and functional neural repair.

**Conclusion:**

The 3D-sEVs@Gel system promotes chronic neurological reconstruction post-TBI by modulating NSC proliferation and neural lineage-associated responses through the CREB1/PGC-1α/FNDC5 pathway, offering a promising cell-free strategy for neural repair and tissue engineering.

## Highlights

• 3D-sEVs@Gel extends retention and enhances long-term functional recovery post-TBI.
• Sustained sEV delivery directs NSC fate and suppresses glial scar formation.
• Exosomal CREB1 activates PGC-1α/FNDC5 pathway to correct aberrant gliogenic fate.

## Graphical abstract

**Figure:**
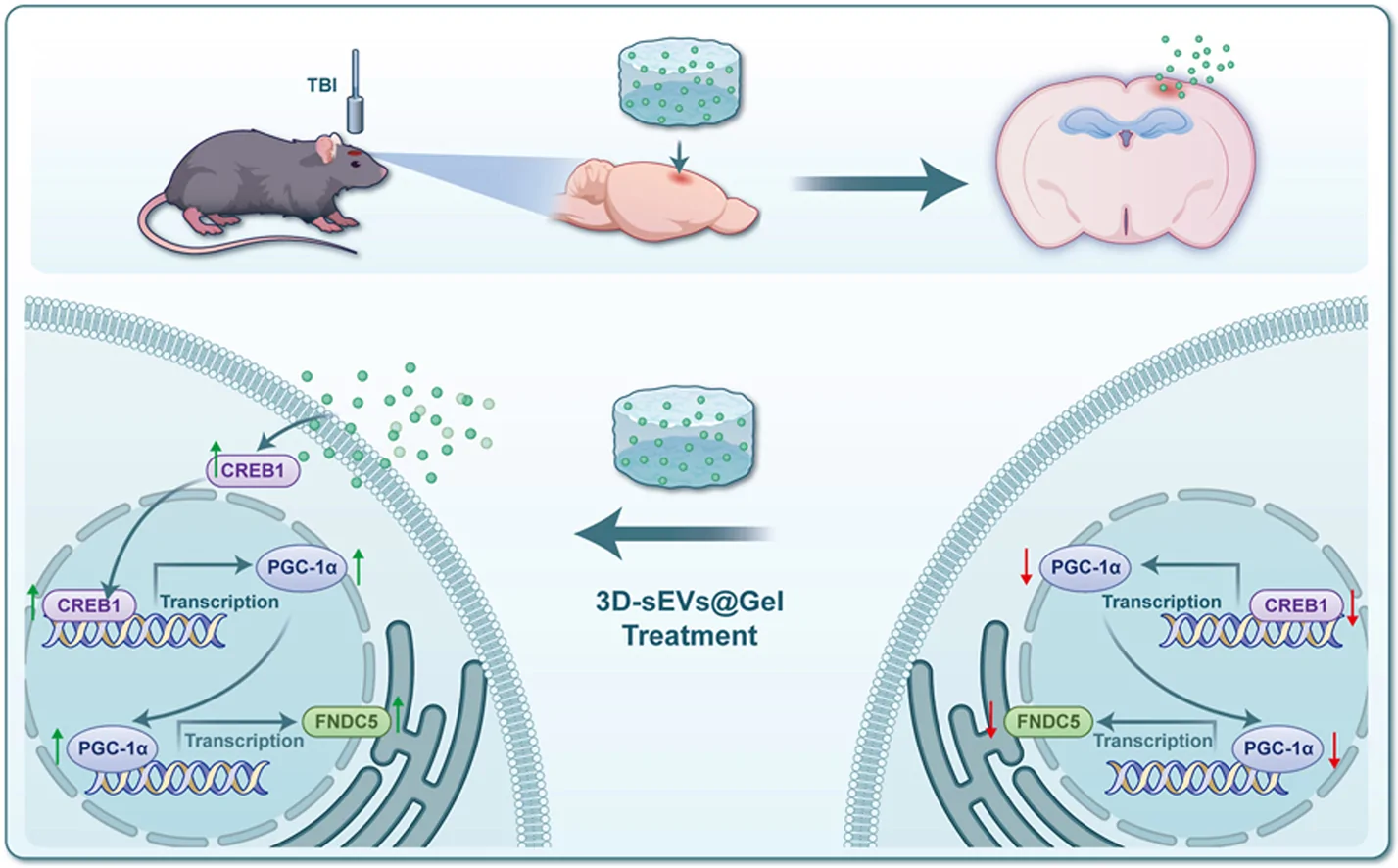

## 1 Introduction

Small extracellular vesicles (sEVs) mediate intercellular communication by transferring bioactive molecules, positioning them as promising therapeutic agents in cell-free regenerative medicine [1], [2]. Under physiological conditions, sEVs maintain tissue homeostasis and promote post-injury repair through paracrine communication [3]. By delivering complex signals derived from stem/progenitor cells, sEVs can simultaneously initiate multiple programs in target tissues, including anti-inflammatory [4], [5], pro-survival, and pro-regenerative effects [6], [7]. This property confers therapeutic potential in central nervous system injuries, particularly in traumatic brain injury (TBI), where the pathological process spans both acute inflammation and chronic repair phases [8], [9].

However, several bottlenecks still impede the clinical translation of sEVs. First, in the upstream production stage, conventional monolayer culture may provide lower yield or different cargo profiles than the present microfiber-based system [10]. Second, in the downstream delivery stage, the *in vivo* half-life of sEVs is only a few hours [11], [12], and the therapeutic window afforded by a single administration is far from sufficient to support chronic tissue repair. In recent years, some 3D bioprinting culture systems have been reported to better recapitulate *in vivo* cell–cell and cell–matrix interactions, thereby significantly enhancing sEV yield and bioactivity [13]. Our previous work has demonstrated that sEVs derived from 3D-bioprinted adipose-derived stem cells (3D-ADSC-sEVs) are superior to 2D-sEVs in terms of cargo loading (e.g., GAS6 protein) and immunomodulatory efficacy [4].

However, the enhancement of intrinsic bioactivity alone does not resolve the issue of rapid *in vivo* clearance of sEVs. Even with highly potent 3D-sEVs, means sEVs generated from ADSCs cultured in the coaxial alginate microfiber system, the failure to extend their half-life would still confine their clinical applications to a narrow therapeutic window spanning merely hours to days. Therefore, the concurrent optimization of both the inherent bioactivity and the *in vivo* residence time of sEVs constitutes a major challenge in the engineering of sEV-based therapeutics.

To address the second bottleneck, biomaterial-mediated local sustained-release systems offer an engineered solution. GelMA hydrogel, owing to its excellent printability, biocompatibility, and tunable degradation kinetics achieved by modulating the crosslinking degree [14], [15], has emerged as an ideal depot platform for localized sEV delivery [16], [17]. Importantly, as demonstrated in previous studies, the physical encapsulation of sEVs within the GelMA network effectively achieves sustained local release [4], [18]. This gradual degradation of the hydrogel not only protects the vesicles from enzymatic clearance but also extends their *in vivo* half-life from a few hours to several days or even weeks [19], [20], [21], [22], [23].

In this study, we addressed the two major clinical challenges of sEVs by employing a 3D bioprinting culture system to generate sEVs with enhanced bioactivity and enriched functional cargo. The resulting sEVs were then incorporated into a GelMA hydrogel to construct a "3D-sEVs@Gel" sustained-release composite system, termed 3D-sEVs@Gel. This design capitalizes on the complementary advantages of both components: the microfiber-based production module was designed to generate sEVs with enhanced cargo loading and activity, while GelMA encapsulation sustains their presence at the lesion site. Our previous work has demonstrated that the 3D-sEVs@Gel system exerts significant anti-inflammatory and neuroprotective effects in the acute phase of a mouse TBI model by delivering GAS6 protein to reprogram microglial phenotypes [4]. However, whether this sustained-release strategy can extend the therapeutic window from the acute phase to the chronic repair phase, thereby driving long-term neurogenesis and functional reconstruction that are unachievable by a bolus injection of 3D-sEVs alone, remains entirely unexplored.

Therefore, in the present study, we systematically evaluated the long-term regenerative effects of the 3D-sEVs@Gel engineered system in a chronic TBI model. Building upon the established sustained-release profile of the GelMA depot and the cargo-loading characteristics observed in sEVs generated by this platform, we hypothesized that this composite system could overcome the temporal limitations of conventional therapies. Indeed, implantation of the 3D-sEVs@Gel system enabled prolonged delivery of these highly potent vesicles. This significantly promoted sustained neurogenesis and structural remodeling. These changes may contribute to the functional recovery induced by 3D-sEVs@GelMA, particularly in spatial learning and memory. Other neuroprotective and tissue-remodeling mechanisms are also likely to be involved. Subsequently, to trace the molecular mechanisms driving these regenerative processes, we integrated transcriptomic and proteomic analyses and revealed that the effects of 3D-sEVs on NSC proliferation and neural lineage-marker expression were associated with CREB1-mediated activation of the downstream PGC-1α/FNDC5 signaling axis (Fig. 1). Ultimately, this study not only establishes a clinically translatable engineering platform for TBI but also provides a comprehensive theoretical framework for harnessing sustained sEV-mediated signaling to overcome the regenerative barriers of the chronic neurological injury microenvironment.

**Fig. 1:**
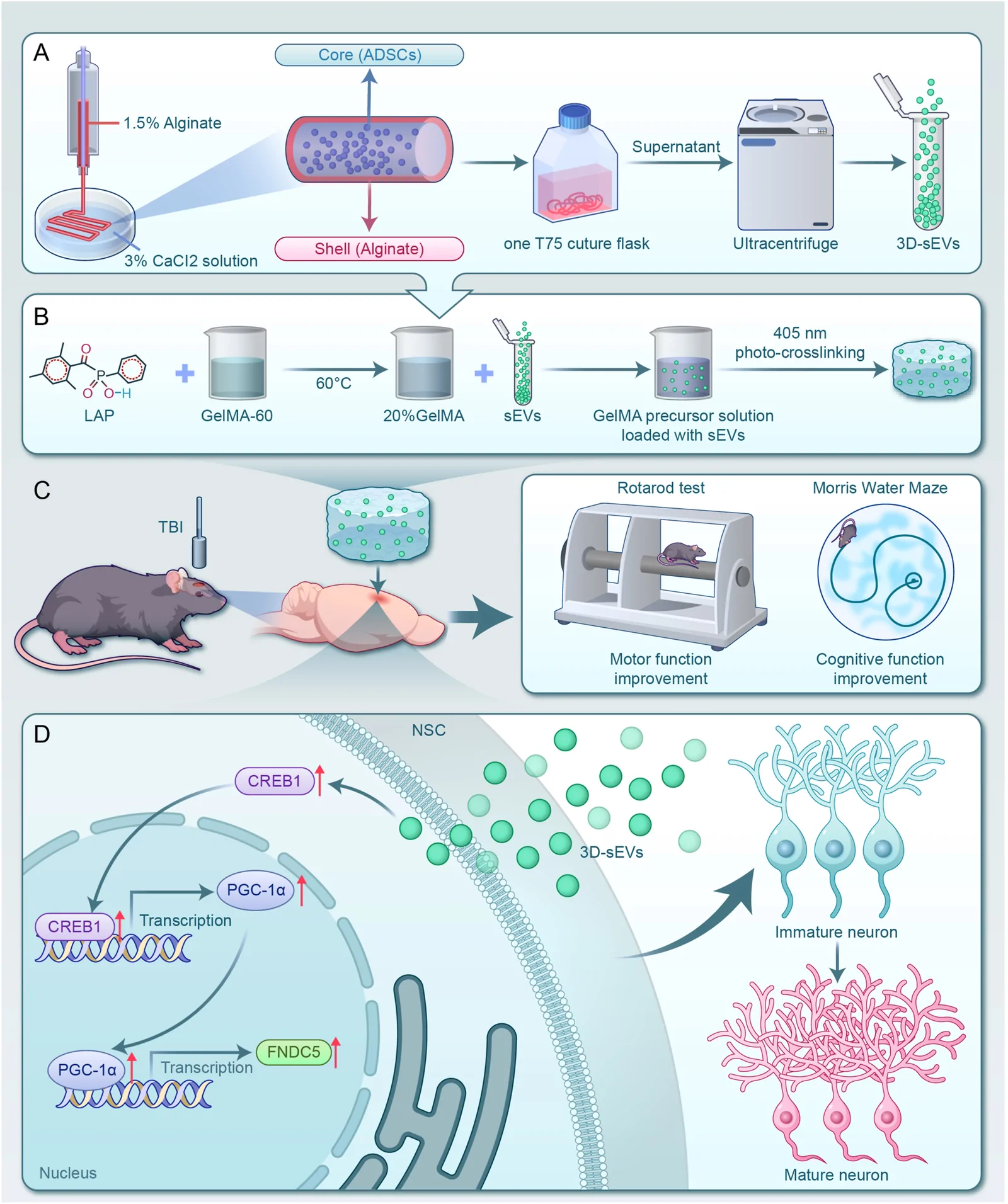
Fabrication and therapeutic mechanisms of the 3D-sEVs@Gel system in TBI repair. (A, B) Schematic of 3D-bioprinted ADSC microfiber culture, sequential ultracentrifugation for 3D-sEV isolation, and formulation of the 3D-sEVs@Gel hydrogel. (C) Orthotopic transplantation of 3D-sEVs@Gel into the mouse TBI lesion and subsequent functional recovery evaluation. (D) Proposed cellular mechanism detailing 3D-sEV uptake by resident NSCs and activation of the CREB1/PGC-1α/FNDC5 signaling axis to promote neurogenesis.

In our previous study, we evaluated the therapeutic effects of the 3D-sEVs@Gel system during the acute phase of TBI using *in vivo* and *in vitro* approaches [4]. Building on that work, the present study focuses on its sustained therapeutic effects during the chronic phase of TBI and on further investigating the mechanisms potentially involved. Accordingly, we first present the long-term *in vivo* outcomes, which address the primary objective of this study, followed by targeted *in vitro* experiments examining the proposed molecular mechanism in NSCs. This organization reflects the primary focus and narrative structure of the present study.

## 2 Materials and methods

### 2.1 Microfluidic engineering of 3D ADSC-encapsulating microfibers

To establish a 3D cell-laden environment for the efficient production of sEVs, ADSC-encapsulating microfibers were fabricated using a coaxial microfluidic spinning technique. Briefly, 3 × 10^8^ ADSCs were uniformly resuspended in 10 mL of complete culture medium to form the core stream. The complete medium was prepared using MesenCult™-ACF Plus Medium (STEMCELL Technologies, Catalog #05445) supplemented with 1× MesenCult™-ACF Plus 500X Supplement (STEMCELL Technologies), 2 mM L-glutamine (Gibco, 25030081), and 1% penicillin-streptomycin (Gibco, 15140122). A 1.5% (w/v) alginate solution was utilized as the sheath fluid. Both fluids were simultaneously extruded through a coaxial nozzle consisting of an inner needle (inner diameter: 0.51 mm, outer diameter: 0.81 mm) and an outer needle (inner diameter: 1.25 mm, outer diameter: 1.61 mm), driven by precision syringe pumps. The flow rates were strictly controlled at 10 mL/h for the core stream and 15 mL/h for the sheath fluid. Continuous microfibers were formed via rapid ionic crosslinking as the fluids were extruded into a 3% (w/v) CaCl_2_ gelation bath. The formed microfibers were immediately collected and washed thoroughly with 0.9% NaCl solution to remove excess calcium ions. Subsequently, the ADSC-laden microfibers were incubated in serum-free medium (NC0103, Yocon, Beijing, China) at 37 °C with 5% CO_2_ for 48 h to generate the conditioned medium. The cell survival status within the microfibers was evaluated using a Live/Dead viability assay kit, and the ultrastructural morphology was characterized by scanning electron microscopy (SEM) following standard fixation and dehydration procedures.

### 2.2 Isolation and characterization of sEVs

For the collection of 2D-sEVs, ADSCs were cultured in monolayers until reaching 60%–80% confluency. After washing with PBS, the cells were incubated in serum-free medium (NC0103, Yocon, China). The conditioned medium was collected every 24 h and pooled until the cells achieved 100% confluency. Concurrently, 3D-sEVs were isolated from the conditioned medium of ADSC microfibers, which was similarly harvested every 24 h and pooled. sEVs were purified from the collected conditioned media via differential ultracentrifugation at 4 °C. The conditioned medium was sequentially centrifuged at 300 × g for 10 min to eliminate dead cells, 2000 × g for 10 min to remove cell debris, and 10,000 × g for 30 min to discard larger microvesicles. The supernatant was then filtered through a 0.22 μm pore filter (JETBIOFIL). Subsequently, the sEVs were pelleted by ultracentrifugation at 100,000 × g for 120 min. The pellet was resuspended in PBS and subjected to a second ultracentrifugation step under the same conditions for washing. The identity and purity of the isolated sEVs were validated according to standard guidelines. Vesicle morphology was examined using transmission electron microscopy (TEM), while size distribution and particle concentration were quantified via nanoparticle tracking analysis (NTA). The presence of sEV-specific canonical markers (CD81, CD63, TSG101) and the absence of the endoplasmic reticulum marker (Calnexin) were confirmed by Western blotting (WB). The final sEV pellets were resuspended in PBS for immediate experimental use or stored at −80 °C. To ensure vesicle integrity, all functional assays were completed within 14 days post-isolation.

### 2.3 Formulation of sEV-Loaded GelMA

To enable the sustained release of sEVs *in vivo*, we formulated a composite hydrogel system by encapsulating sEVs into GelMA (Suzhou Yongqinquan Intelligent Equipment Co., Ltd., China, EFL-GM-60), designated as sEVs@Gel. Briefly, a 0.5% (w/v) photoinitiator LAP solution was prepared and mixed with GelMA (EFL, China) at a 5:1 mass ratio. The mixture was heated to 60 °C with intermittent vortexing to ensure complete dissolution, yielding a 20% (w/v) GelMA precursor solution. This precursor was subsequently sterilized by passing it through a 0.22-μm syringe filter and cooled to 37 °C. For sEV encapsulation, a highly concentrated sEV suspension (5 × 10^11^ particles/mL in sterile PBS) was gently mixed with an equal volume of the sterile 20% GelMA solution. The mixture was incubated on an orbital shaker at 20 rpm and 37 °C for 30 min to ensure uniform dispersion, resulting in a final GelMA concentration of 10% (w/v) in the sEV-laden precursor. Finally, 10 μL of the sEV-laden precursor solution was pipetted into customized cylindrical molds (2 mm in diameter and 3 mm in height). Gelation was induced by photo-crosslinking under a 405-nm light source with an intensity of 110 mW/cm^2^ for 30 s. Pure GelMA hydrogels (without sEVs) were prepared following the identical procedure using an equal volume of sterile PBS instead of the sEV suspension.

### 2.4 TBI model

Male C57/BL6J mice, aged 12 weeks, were obtained from Hunan Slack Laboratory Animal Co., Ltd. (Hunan, China). All animals were housed in the animal facility of the Laboratory Animal Research Center at Central South University under specific pathogen-free conditions, with controlled temperature and humidity, a 12-h light/dark cycle, and environmental enrichment. Food and water were accessible ad libitum. All animal experiments were conducted in strict accordance with the guidelines established by the Institutional Animal Care and Use Committees of Central South University, Hunan, China (Approval No. 2023030529). The controlled cortical impact (CCI) model was established according to the protocol described in our previous studies [4], [23]. Briefly, following induction of anesthesia with 5% isoflurane and maintenance with 1.5% isoflurane, the dorsal scalp of the mouse was shaved and disinfected. A midline incision was made to expose the calvarial vault. A burr hole with a diameter of 5 mm was then drilled at a designated location (right parietal bone, centered 3 mm posterior to the bregma and 2 mm lateral to the midline), with care taken to avoid damage to the underlying brain parenchyma. Subsequently, a flat-tipped impactor was applied using the following parameters: velocity 5.0 m/s, penetration depth 2.0 mm, and dwell time, 0.15 s. Immediately after impact, hemostasis was carefully performed at the lesion site. After hemostasis was achieved, equal volumes of PBS, GelMA, 2D-sEVs@Gel, or 3D-sEVs@Gel were implanted into the lesion site, followed by suturing of the skin incision. Structural and functional assessments were conducted at 14 days post-injury (subacute phase) and 28 days post-injury (chronic phase).

### 2.5 Preparation of brain tissue sections

On days 14 and 28 post-CCI, mice were deeply anesthetized with inhaled isoflurane and then humanely euthanized according to the approved animal protocol. The mice were subsequently placed in a supine position on an operating board. The thoracic cavity was opened to expose the heart, and the right atrial appendage was incised. PBS was perfused through the left ventricle, followed by fixation with 4% paraformaldehyde (PFA). After perfusion, the brain was carefully removed and post-fixed in 4% PFA at 4 °C in the dark for 24 h. Subsequently, the brain tissue was dehydrated by sequential immersion in 20%, 30%, and 35% sucrose-PBS solutions at 4 °C until the tissue completely sank to the bottom. After dehydration, the brain was removed, and excess liquid was blotted dry. The brain was then completely embedded in pre-chilled OCT compound (4583, Sakura) in a dry ice or liquid nitrogen environment and rapidly frozen into solid blocks. The tissue block was fixed onto the specimen holder of a cryostat (CM1950, Leica), and coronal sections centered on the lesion core (TBI core region) were collected, covering the lesion area, the subventricular zone (SVZ), and the hippocampal dentate gyrus (DG) region. The section thickness was set to 20 μm. The sections were flattened and adhered onto adhesive microscope slides, air-dried at room temperature for 30 min, and then stored in a −80 °C ultra-low-temperature freezer for long-term preservation before subsequent histomorphological observation and immunofluorescence staining analysis.

### 2.6 Internalization of sEVs *in vivo* and *in vitro*

To track the cellular uptake of sEVs, purified ADSC-derived sEVs were labeled with the lipophilic membrane dye DiI (V22885, Thermo Fisher Scientific, USA). The sEVs were co-incubated with 1 μM DiI dye at 37 °C for 30 min in the dark. Unincorporated dye was subsequently removed by ultracentrifugation at 100,000 × g for 70 min, and the DiI-labeled sEVs were resuspended in sterile PBS. For the *in vitro* cellular uptake assay, the cultured NSCs (prepared as described in Section 2.7) were seeded onto laminin-coated coverslips. DiI-labeled sEVs (1 × 10^11^ particles/mL) were added to the culture medium and co-incubated for 4 h at 37 °C. Post-incubation, the cells were washed with PBS, fixed with 4% PFA for 15 min, and counterstained with DAPI for 5 min to visualize the nuclei. The internalization was evaluated using a confocal laser scanning microscope (N2-DM4B, Leica). For the *in vivo* internalization assessment, the DiI-labeled sEVs were encapsulated into GelMA hydrogels to formulate DiI-sEVs@Gel, strictly following the photopolymerization protocol (using 405-nm light) described in Section 2.3. The DiI-sEVs@Gel constructs were then orthotopically implanted into the lesion cavity of the CCI mouse model, as detailed in Section 2.4. On day 14 post-implantation, the mice were euthanized and perfused, and 20-μm-thick coronal frozen sections were prepared (Section 2.5). The co-localization of DiI-sEVs and SOX-2-positive NSCs within the perilesional area was ultimately visualized and captured using the confocal microscope.

To investigate the uptake efficiency of sEVs by NSCs *in vitro*, both 2D-sEVs and 3D-sEVs were labeled with DiI. Subsequently, equal volumes of DiI-labeled 2D-sEVs or 3D-sEVs were added to the medium of NSCs and co-incubated for 6 h. After incubation, immunofluorescence staining was performed to label SOX-2, and co-localization of DiI-sEVs with SOX-2 was observed using microscopy.

### 2.7 NSCs treatments

Having established the long-term effects of 3D-sEVs@GelMA *in vivo*, we next performed targeted *in vitro* experiments to investigate the molecular mechanisms that may contribute to the observed biological responses. In the neural differentiation experiment, NSCs were incubated with fresh culture medium (negative control) or fresh culture medium containing 2D-sEVs or 3D-sEVs for 24 h. Subsequently, NSCs were harvested for WB, real-time quantitative polymerase chain reaction (RT-qPCR), scratch assay, Transwell assay, or immunofluorescence staining. In the mechanistic study, the experimental groups included PBS, PBS + CREB1 inhibitor, 3D-sEVs, and 3D-sEVs + CREB1 inhibitor. NSCs were treated with the specific CREB1 inhibitor 666-15 (50 nM, MedChemExpress, 1433286-70-4), while 3D-sEVs were administered at a concentration of 2 × 10^9^ particles/mL. After 24 h of incubation, NSCs were collected for subsequent RNA extraction, WB, or immunofluorescence staining.

### 2.8 Transcriptomic sequencing and analysis

Transcriptomic sequencing and analysis were performed by Beijing Youji Technology Co., Ltd. Gene-level raw counts were generated using featureCounts, and FPKM (Fragments Per Kilobase of transcript per Million mapped reads) values were calculated for expression visualization. Differentially expressed genes (DEGs) among the Control, 2D-sEVs, and 3D-sEVs groups were identified using DESeq2. Genes with Padj < 0.05 and |log10FC| > 1 were considered differentially expressed. GO enrichment analysis was performed using clusterProfiler and org.Mm.eg.db, and GSEA was conducted using msigdbr and fgsea. Detailed procedures are provided in the Supplementary Methods.

### 2.9 Proteomic analysis of ADSC-sEVs

To systematically characterize the protein composition of 2D-sEVs and 3D-sEVs, label-free quantitative proteomic analysis was performed by Wayen Biotechnologies (Shanghai) Co., Ltd. Briefly, sufficient quantities of ADSC-sEVs were obtained via ultracentrifugation and subsequently submitted for protein extraction. The total sEV proteins were then reduced, alkylated, and digested into peptides using trypsin. Subsequently, the purified peptide mixtures were separated by high-performance liquid chromatography (HPLC) and analyzed using a tandem mass spectrometer (LC-MS/MS). Finally, the generated raw mass spectrometric data were processed and searched against the relevant *Homo sapiens* UniProt database for protein identification and relative label-free quantification. Detailed protocol for proteomic analysis is provided in the Supplementary Methods.

### 2.10 Generation of CREB1-depleted ADSC-derived 3D-sEVs

ADSCs were transduced with GFP-expressing lentiviral vectors carrying one of four CREB1-targeting shRNAs (sh-CREB1-1 to sh-CREB1-4) or a non-targeting control shRNA (sh-NC) (Suzhou GenePharma Co., Ltd) at a multiplicity of infection (MOI) of 50, according to the manufacturer's instructions. Transduction was monitored by GFP fluorescence. CREB1 knockdown was screened by Western blotting and qRT-PCR, and the construct showing the greatest knockdown efficiency (sh-CREB1-2) was selected for subsequent experiments. The transduced ADSCs were then cultured using the coaxial 3D bioprinting culture system described in Section 2.1. Conditioned medium was collected and processed for sEV isolation by ultracentrifugation as described in Section 2.2. The isolated preparations were examined by TEM and characterized by Western blotting for CREB1, TSG101, CD63, CD9, and Calnexin. CREB1, FNDC5, and PGC-1α levels were also assessed in the sEV preparations.

### 2.11 Treatment of NSCs with CREB1-depleted 3D-sEVs

NSCs were cultured as described in Section 2.7 and treated with 3D-sEVs, 3D-sEVs^sh-NC^, or 3D-sEVs^sh-CREB1-2^ at an equivalent dose of 5 μg/mL for 48 h. After treatment, cells were collected for Western blotting or fixed for immunofluorescence analysis. Western blots were performed to assess DCX, MAP-2, GFAP, and Olig-2 expression, with β-actin used as the loading control.

### 2.12 Statistics

All quantitative data are presented as the mean ± standard error of the mean (SEM) from at least three independent biological replicates. Statistical analyses and graphical representations were generated using GraphPad Prism software (version 9.0.0, GraphPad Software, San Diego, CA, USA). Prior to hypothesis testing, the normality of the data distribution was assessed using the Shapiro-Wilk test, and the homogeneity of variances was confirmed by Levene's test. For data adhering to a normal distribution, comparisons between two independent groups were performed using the unpaired Student's t-test. For comparisons involving three or more experimental groups with a single independent variable (e.g., *in vitro* functional and mechanistic assays), a one-way analysis of variance (ANOVA) followed by Tukey's post-hoc test for multiple comparisons was utilized. To evaluate the dynamic behavioral alterations over time (i.e., Morris Water Maze learning curves and rotarod test latency), a two-way repeated-measures ANOVA followed by Bonferroni's post-hoc test was applied to determine main effects and interactions between time points and treatment groups. For non-normally distributed data, the non-parametric Kruskal-Wallis test followed by Dunn's multiple comparisons test was adopted. Statistical significance was defined as P < 0.05. The specific levels of significance are denoted in the figures and corresponding legends as *P < 0.05, **P < 0.01, and ***P < 0.001.

### 2.13 Other methods

Detailed descriptions of additional methodological procedures are provided in the Supporting Information. These encompass primary cell handling (collection and culture of ADSCs, isolation and culture of NSCs); biocompatibility evaluation of GelMA hydrogels; functional cellular assays (NSC proliferation and Transwell migration); molecular and biochemical analyses (RT-qPCR, Western blot, ELISA, and immunofluorescence staining); as well as *in vivo* histological and functional assessments (evaluation of brain tissue loss and behavioral testing).

## 3 Results

### 3.1 Coaxial bioprinting and in vitro culture of ADSCs

First, after expanding ADSCs to the fifth passage, a total of 3 × 10^8^ ADSCs were coaxially bioprinted using a core–shell bioprinting technique [4]. The ADSC-laden microfibers were then transferred into culture flasks for further cultivation (Fig. 1A). Subsequently, optical microscopy, live/dead assays, and SEM were employed to evaluate the viability and growth behavior of ADSCs within the coaxially bioprinted microfibers. The 3D bioprinting culture system exhibited a highly organized, interconnected 3D porous network (Fig. 2A). Live/dead staining demonstrated that ADSCs maintained high viability even after 7 days of culture without passaging (Fig. 2B). Scanning electron microscopy (SEM) analysis further confirmed the microstructure, revealing a porous architecture enveloped by a continuous alginate sheath, thereby creating a suitable microenvironment for intercellular communication and dynamic cell–matrix interactions (Fig. 2C).

**Fig. 2:**
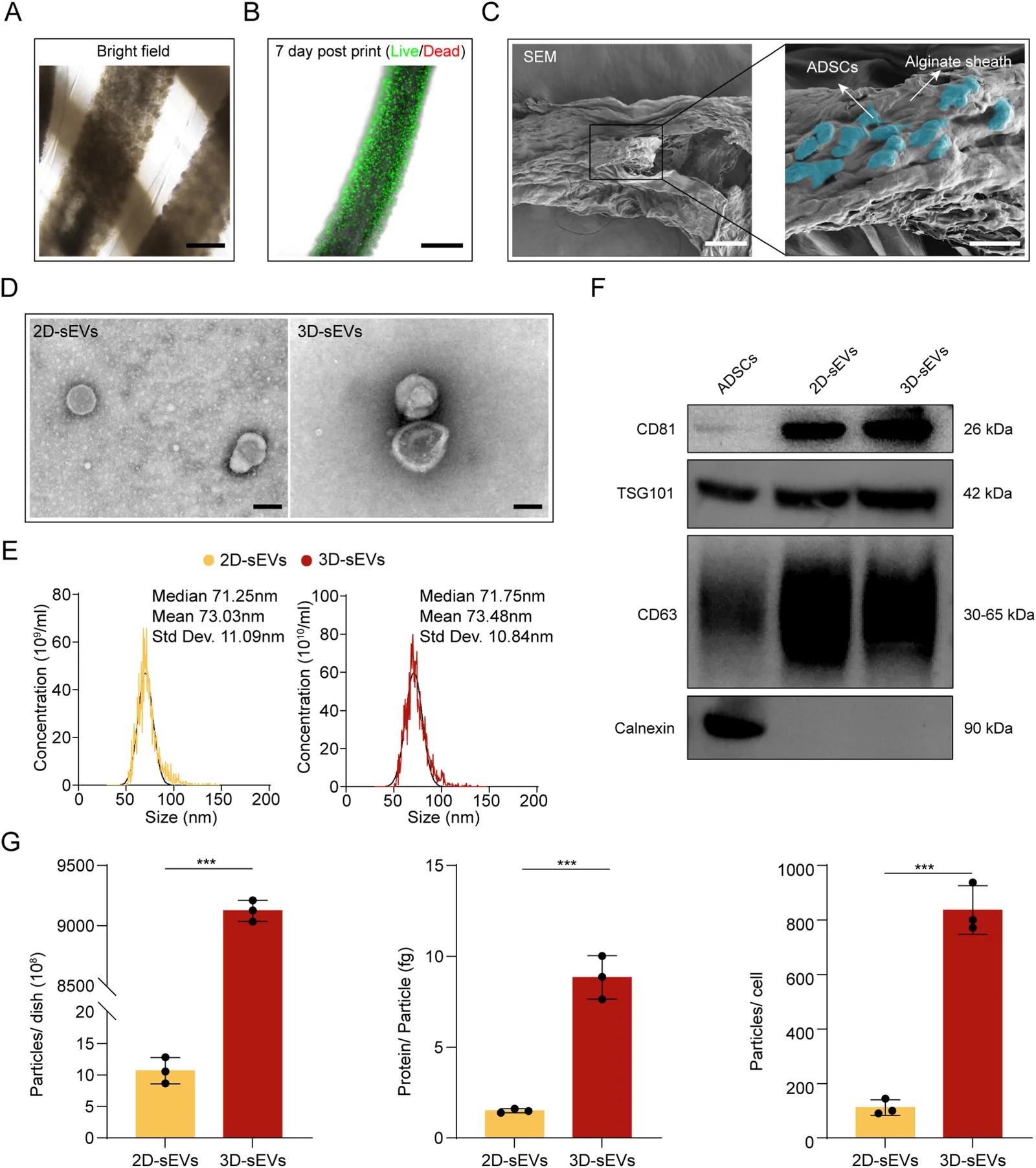
Characterization of ADSC-laden alginate microfibers and sEVs derived from 2D- and 3D-cultured ADSCs. (A) Representative bright-field image of the printed microfibers (scale bar = 500 μm). (B) Representative Live/Dead staining image of the microfibers 7 days after printing; live cells are shown in green and dead cells in red (scale bar = 500 μm). (C) SEM image of the microfiber surface and a magnified view; the cyan overlay identifies ADSCs within the alginate sheath (scale bar = 200 μm (low magnification) and 30 μm (high magnification)). (D) Representative TEM images of 2D-sEVs and 3D-sEVs (Scale bars = 100 nm). (E) NTA size-distribution profiles of 2D-sEVs and 3D-sEVs; median, mean, and standard deviation are indicated. (F) Western blot analysis of CD81, TSG101, CD63, and calnexin in ADSC lysates and isolated sEVs. (G) Comparison of particle yield per dish, protein yield per dish, and particle yield per cell for 2D-sEVs and 3D-sEVs. Data are presented as mean ± SD (n = 3). ***P < 0.001.

### 3.2 Comparative characterization of sEVs derived from 3D and 2D cultured ADSCs

Next, sEVs were isolated from the conditioned medium of ADSCs cultured in 3D microfibers or in a 2D system using sequential ultracentrifugation (Fig. 1A). Subsequently, the sEVs from these two different sources were characterized using TEM, Western blot (WB), and nanoparticle tracking analysis (NTA). TEM analysis confirmed that both 2D-sEVs and 3D-sEVs exhibited a double-membrane morphology and a cup-shaped structure (Fig. 2D). NTA analysis revealed that the sEVs derived from the 3D bioprinting culture system exhibited a mean size of 73.48 ± 10.84 nm, which was within the typical exosomal size range and comparable to that of the 2D culture system (Fig. 2E). WB results demonstrated positive expression of exosomal marker proteins (CD63, TSG101, and CD81) and negative expression of the endoplasmic reticulum marker protein Calnexin in the isolates, confirming that they met the basic characteristics of sEVs (Fig. 2F, Fig. S3). Quantitative analysis (Fig. 2G) further revealed that, compared with 2D monolayer culture (approximately 100 sEVs per cell), coaxial alginate microfiber 3D bioprinting culture system not only significantly increased the number of sEVs secreted per cell (approximately 800 sEVs per cell) by approximately 8-fold, but also achieved a dramatic increase in the total output per culture dish, reaching approximately 9120 × 10^8^ particles per dish. Moreover, the protein abundance per individual 3D-sEV (8–9×10^−6^ ng/particle) was significantly higher than that of 2D-sEVs (1.5 × 10^−6^ ng/particle). The present coaxial alginate-microfiber culture system was associated with increased sEV yield and protein content per particle compared with monolayer culture.

To determine whether sEV origin influenced GelMA-mediated release, we compared the *in vitro* release profiles of 2D-sEVs@GelMA and 3D-sEVs@GelMA under matched hydrogel preparation and loading conditions. BCA-based quantification showed similar cumulative protein-release profiles for the two formulations, with approximately 80% of the initially loaded protein recovered in the release medium by day 16. No statistically significant difference was detected between the groups under the conditions tested (Fig. S4).

### 3.3 3D-sEVs@Gel orchestrates multi-regional neural regeneration and functional recovery after TBI

We subsequently evaluated the long-term neurorestorative and neuroregenerative efficacy of 3D-sEVs in a mouse model of TBI. Based on our previous study [4], 2D-sEVs or 3D-sEVs at a concentration of 5 × 10^11^ particles/mL were loaded into 20% GelMA hydrogels (designated as 2D-sEVs@Gel and 3D-sEVs@Gel, respectively) (Fig. 3B). Following TBI, GelMA alone, 2D-sEVs@Gel, and 3D-sEVs@Gel were orthotopically transplanted into the injury site immediately (Fig. 3, Fig. 4A). To comprehensively evaluate functional recovery following TBI, the modified Neurological Severity Score (mNSS) and rotarod test were employed to assess general neurological deficits and sensorimotor coordination (Fig. 3A–D), respectively, while the Morris water maze (MWM) was utilized to evaluate spatial learning and cognitive memory (Fig. 4A–B). The results showed that 3 days after injury, the latency to fall in all mice subjected to CCI dropped sharply, from a pre-injury baseline of 260-290 s to below 50 s, and the 3D-sEVs@Gel group exhibited the most significant and sustained upward trend (Fig. 3E). At 14 days post-TBI, the mean latency to fall in the 3D-sEVs@Gel group (approximately 200 s) was significantly higher than that in the 2D-sEVs@Gel group (approximately 100-120 s) and other treatment groups (Fig. 3F). During the chronic phase (days 28–35 post-TBI), the 3D-sEVs@Gel group maintained a significantly prolonged falling latency (approximately 245–250 s) compared to the 2D-sEVs@Gel and vehicle groups (Fig. 3E), demonstrating robust and sustained long-term motor reconstruction. The Morris water maze test showed a similar pattern. On day 28 post-TBI, mice in the 3D-sEVs@Gel group exhibited a shorter and more goal-oriented path to locate the hidden platform and displayed a highly focused search pattern in the target quadrant (Fig. 4C). The learning curve over the five consecutive days of the water maze test (Fig. 4D) showed that the escape latency decreased progressively with training in all groups; however, the learning acquisition rate in the 3D-sEVs@Gel group was markedly superior to that in the other intervention groups. By day 5 of training (Fig. 4E), the mean escape latency in the 3D-sEVs@Gel group was reduced to 18.4 ± 3.2 s, which was significantly shorter than that in the 2D-sEVs@Gel group (31.5 ± 4.1 s) and the scaffold-only group (45.8 ± 6.2 s) (P < 0.001). The mNSS results on day 28 (Fig. 4H) indicated that sEV intervention significantly improved neurological functional performance in mice, as reflected by lower mNSS scores. Among them, the 3D-sEVs@Gel treatment group exhibited the most pronounced recovery of neurological function, with an mNSS score of 5.3 ± 0.8, which was significantly better than that of the 2D-sEVs@Gel treatment group (9.2 ± 1.1, P < 0.05). Among the sEV@Gel groups, the 3D-sEVs@Gel group consistently exhibited the lowest tissue-loss rate at both the subacute and chronic phases, with a quantified value of 18.08% ± 1.39% at 14 days post-injury (Fig. 3G), and this structural preservation was sustained through 28 days as confirmed by Nissl staining (Fig. 4F, Fig. S5A). Higher-magnification Nissl-stained images further revealed that CCI reduced the number and organization of Nissl-positive neuronal profiles and increased neuronal morphological abnormalities in the peri-lesional cortex (Figs. S6–7) and hippocampal CA1, DG, and CA3 regions (Figs. S6–7). These changes were partially improved by 2D-sEVs@GelMA and more prominently by 3D-sEVs@GelMA.

**Fig. 3:**
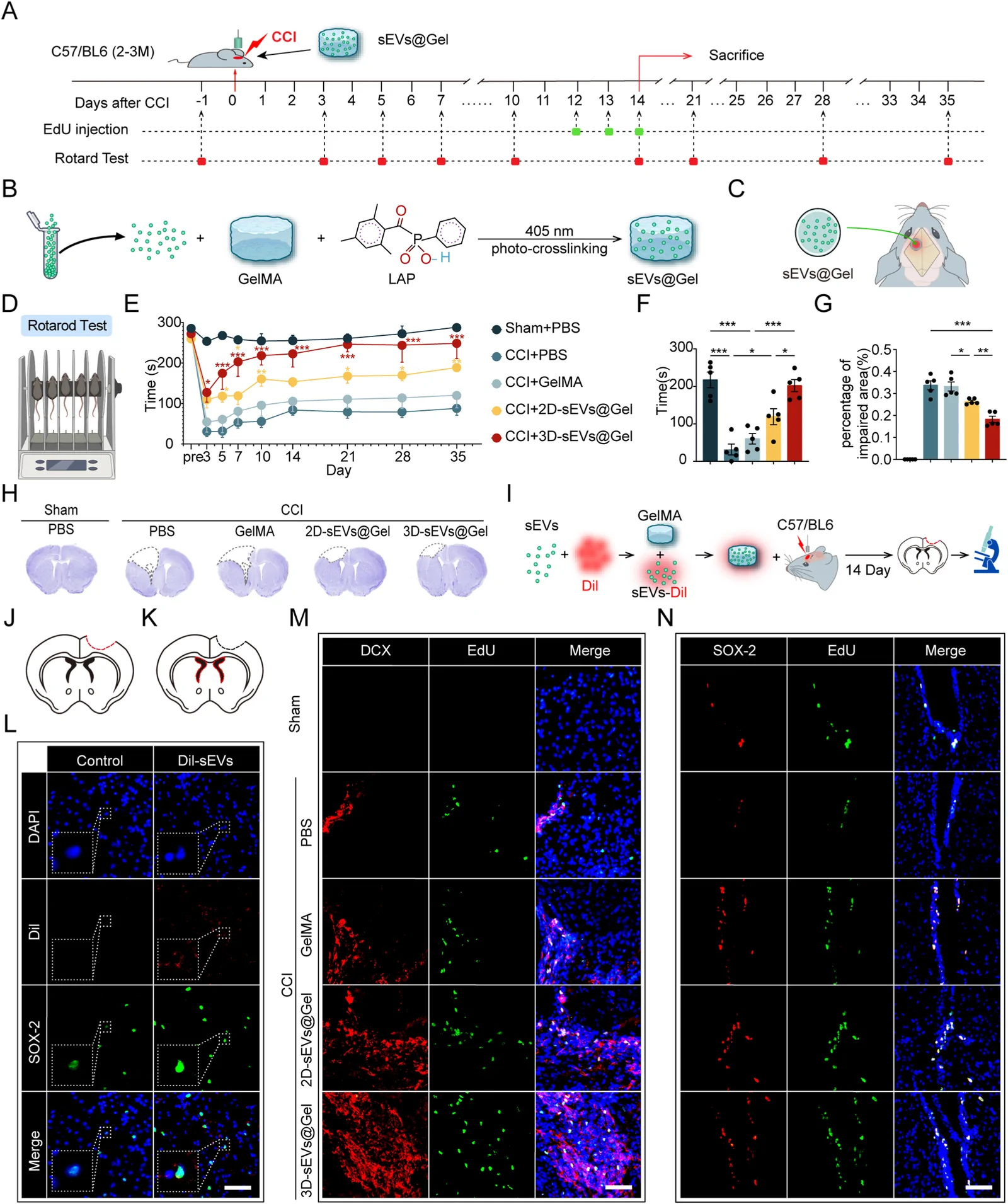
Therapeutic efficacy and neuroregenerative outcomes of 3D-sEVs@Gel at 14 days post-TBI (subacute phase). (A) Experimental timeline outlining CCI induction, scaffold implantation, EdU labeling, behavioral testing, and endpoint evaluation (day 14). (B, C) Schematics illustrating (B) sEV encapsulation via photo-crosslinked GelMA hydrogels and (C) local implantation onto the lesion site. (D–F) Rotarod apparatus (D) and motor coordination performance over 35 days (E), with detailed comparison at day 14 (F). (G, H) Quantification of brain tissue deficit (G) and representative Nissl-stained brain sections (H; dashed lines indicate ventricular enlargement). (I–K) Schematics showing DiI-sEV tracking (I), implant location (J), and target analytical regions (K). (L) Confocal images confirming colocalization of DiI-sEVs (red) with SOX-2^+^ progenitor cells (green) at day 14 (Scale bars: 100 μm). (M, N) Confocal images showing increased numbers of EdU-labeled cells co-expressing the immature neuronal marker DCX (M) and the neural stem/progenitor-associated marker SOX-2 (N) in the 3D-sEVs@Gel group. Data are mean ± SD (***p < 0.001), scale bars: 100 μm.

**Fig. 4:**
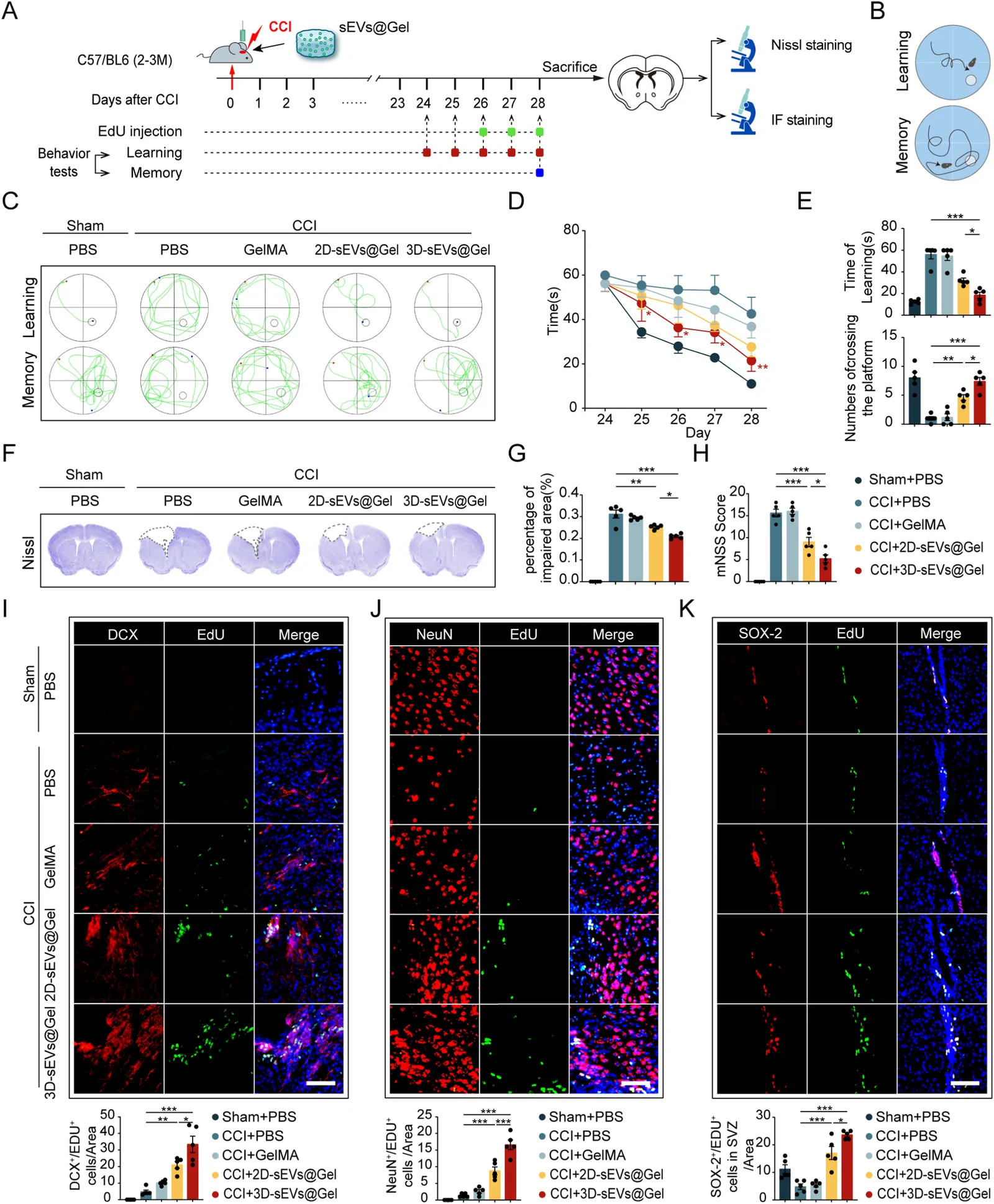
Long-term therapeutic efficacy and endogenous neurogenesis promoted by 3D-sEVs@Gel at day 28 post-TBI. (A) Experimental timeline for chronic phase behavioral (MWM), proliferation tracking (EdU), and histological evaluations. (B) MWM apparatus schematic. (C–E) Representative swimming trajectories (C), escape latency curves during training (D), and quantifications of learning duration and target platform crossings during the probe trial (E). (F, G) Representative Nissl-stained coronal sections (F; dashed lines outline lesion boundaries) and lesion area percentage quantification (G). (H) mNSS scores at 28 days after TBI. Lower scores represent better functional recovery. Data are mean ± SEM (n = 5). (I–K) Representative confocal images and cell density quantifications for EdU-labeled cells co-expressing DCX, NeuN, or SOX-2, representing immature neuronal-, mature neuronal-, and neural stem/progenitor-associated phenotypes, respectively. Data are mean ± SD (*p < 0.05, **p < 0.01, ***p < 0.001), scale bar: 100 μm.

For *in vivo* tracking, 3D-sEVs were pre-labeled with the fluorescent dye DiI (Fig. 3I). Immunofluorescence imaging at 14 days post-injury revealed distinct DiI signals retained within the peri-lesion area (Fig. 3L). Notably, these signals were extensively internalized by SOX-2^+^ neural stem/progenitor cells (Fig. 3L), confirming successful vesicle retention and cellular uptake. We further assessed neurogenesis after TBI. DCX^+^/EdU^+^ double staining results showed that, in the subacute phase (Fig. 3M, Fig. S8B), the CCI+3D-sEVs@Gel group exhibited the highest number of DCX^+^/EdU^+^ cells (23.6 ± 2.86), and these cells exhibited more complex neurite structures; in the chronic phase (Fig. 4I), the number of DCX-positive cells in the peri-lesional area was significantly higher in the CCI+3D-sEVs@Gel group than in other groups. Pearson correlation analysis (Fig. S8C, Fig. S5B, E) revealed that a greater number of newborn neurons in the injured brain was associated with better recovery of motor coordination and cognitive memory functions. In the chronic phase (Fig. 4J), the 3D-sEVs@Gel group exhibited a significantly higher number of EdU-labeled cells co-expressing NeuN than the other groups (16.60 ± 1.47). Pearson correlation analysis (Fig. S5C and F) indicated that a higher number of mature neurons in the injured brain correlated with better recovery of motor coordination function. Collectively, these marker-based findings are consistent with enhanced proliferation and neuronal-lineage-associated responses following 3D-sEVs@Gel treatment; however, they do not establish the lineage origin of the labeled cells.

Furthermore, in the subacute phase following TBI, abundant co-localization signals of SOX-2 and Olig-2 were observed in the CCI+3D-sEVs@Gel group (Fig. S9A–B), indicating that 3D-sEVs significantly increased the number of cells co-expressing SOX-2 and the oligodendroglial-lineage-associated marker Olig-2. Pearson correlation analysis revealed a statistically significant association between the increase in SOX-2^+^/Olig-2^+^ cells and the improvement in motor function in mice. In the chronic phase following TBI, a trend toward an increased number of SOX-2^+^/Olig-2^+^ cells was also observed (Fig. S10A–C).

Interestingly, compared with other groups, the 3D-sEVs@Gel group exhibited a significantly higher number/density of SOX-2^+^/EdU^+^ cells within the SVZ and the hippocampal DG—two canonical neurogenic niches [24]—during both the subacute (Fig. 3N, Fig. S8B, Fig. S9C) and chronic (Fig. 4K, Fig. S10D) phases following TBI. This finding suggests that the ability of 3D-sEVs to promote newborn neuron regeneration is not limited to the local injury site; rather, the active cargoes released by 3D-sEVs may promote proliferative responses among cells expressing neural stem/progenitor-associated markers within these neurogenic niches through cerebrospinal fluid circulation or intercellular signaling. Pearson correlation analysis showed an association between the densities of neural lineage-marker-positive cells and behavioral improvement in motor coordination observed in behavioral tests, suggesting that multi-regional neurogenesis may be one of the underlying mechanisms contributing to neurological recovery in both the subacute (Fig. S8D, Fig. S9D) and chronic phases (Figs. S5D, G, Fig. S10E-F).

To further determine whether the behavioral improvements were accompanied by structural neural remodeling, we examined GAP-43 and MAP-2 immunoreactivities in the peri-lesional cortex at 14 and 28 days after CCI. At day 14, both GAP-43 and MAP-2 signals were reduced in the CCI + GelMA group compared with the Sham group. Treatment with 2D-sEVs@GelMA partially restored these signals, whereas 3D-sEVs@GelMA produced a more pronounced recovery (Fig. S11). These effects persisted at day 28 (Fig. S12). Furthermore, GAP-43 and MAP-2 immunoreactivities were positively associated with rotarod performance at day 14 and with Morris water maze performance at day 28. These results indicate that the functional benefits of sEV@GelMA treatment are accompanied by enhanced axonal growth-associated responses and dendritic structural remodeling.

### 3.4 3D-sEVs modulate NSC proliferation, migration, and neural lineage-associated differentiation in vitro

Subsequently, the reparative effects of 3D-sEVs on NSCs were validated through *in vitro* experiments. First, DiI-labeled sEVs were found to be effectively internalized by Nestin^+^ NSCs regardless of the culture mode (Fig. 5A–C). In terms of cell migration, the Transwell assay and scratch assay revealed that NSCs treated with 3D-sEVs exhibited enhanced migratory capacity (Fig. S13A–C). Subsequently, the regulatory effects of 3D-sEVs on NSC differentiation were validated using WB, RT-qPCR, and immunofluorescence assays (Fig. 5B). RT-qPCR analysis (Fig. 5D) revealed that, compared with the 2D-sEVs group, 3D-sEVs treatment significantly upregulated the mRNA expression of the oligodendrocytic marker Olig-2, the mature neuronal marker MAP-2, and the immature neuronal marker DCX, while downregulating the mRNA expression of the astrocytic marker GFAP. Western blot results (Fig. 5E–F) corroborated the above findings. Furthermore, immunofluorescence analysis further supported changes in NSC proliferation and neural lineage-marker expression and microenvironmental remodeling in the 3D-sEVs-treated group (Fig. 5G–J). Specifically, the treatment significantly increased the proportion of SOX-2^+^/EdU^+^ cells (Fig. 5G), indicating the robust activation and proliferation of NSCs. This was closely coupled with a prominent rise in DCX^+^/EdU^+^ cells (Fig. 5H), indicating an increased number of proliferating cells expressing the immature neuronal marker DCX. Concurrently, a substantial expansion of mature neurons (MAP-2^+^) was observed alongside a marked reduction in reactive astrocytes (GFAP^+^) (Fig. 5I), indicating a shift in marker expression toward a more neuronal and less astrocytic phenotype. Furthermore, a large population of SOX-2^+^/Olig-2^+^ cells emerged in the 3D-sEVs group (Fig. 5J), revealing that the treatment additionally increased the number of SOX-2^+^/Olig-2^+^ cells, consistent with enhanced oligodendroglial-lineage-associated differentiation.

**Fig. 5:**
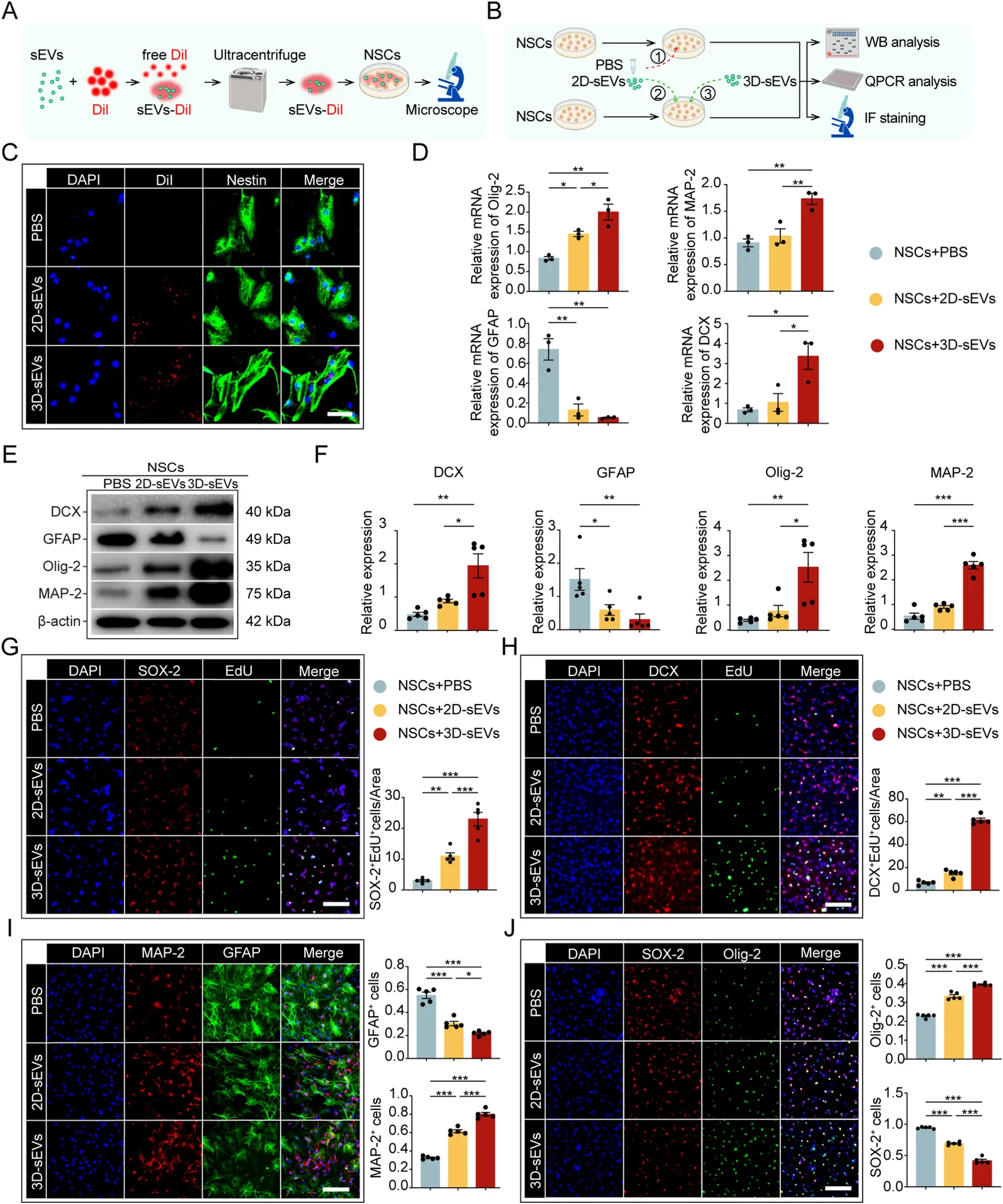
*In vitro* sEV uptake and regulation of NSC proliferation and multi-lineage differentiation. (A, B) Schematics illustrating DiI-sEV uptake protocol (A) and overall *in vitro* experimental workflow (B). (C) Confocal images confirming uptake of DiI-sEVs (red) by Nestin^+^ NSCs (green), scale bar: 50 μm. (D) RT-qPCR analysis of lineage marker mRNA expression (Olig-2, MAP-2, GFAP, and DCX). (E, F) Representative Western blot (E) and densitometric quantification (F) of lineage marker protein levels (β-actin loading control). (G–J) Representative IF images and quantifications evaluating NSC proliferation (SOX-2^+^/EdU^+^, G), neuroblast proliferation (DCX^+^/EdU^+^, H), neuronal versus astrocytic differentiation (MAP-2^+^ vs. GFAP^+^, I), and oligodendroglial-lineage-associated marker co-expression (SOX-2^+^/Olig-2^+^, J). Data are mean ± SD (*p < 0.05, **p < 0.01, ***p < 0.001), scale bar: 100 μm.

### 3.5 Transcriptomic and proteomic analyses identify FNDC5 as a key mediator of 3D-sEVs-induced neurogenesis

To elucidate the molecular mechanisms underlying 3D-sEV-mediated neural regeneration and functional recovery after TBI, we integrated transcriptomic and proteomic analyses. Differential analysis (Fig. 6A) revealed that, compared with 2D-sEVs, 3D-sEVs induced more extensive transcriptomic changes (Fig. S14–S16). GO enrichment analysis showed that the DEGs upregulated following 3D-sEV intervention were highly enriched in key biological processes, including "regulation of neurogenesis", "regulation of developmental growth", and "axonogenesis" (Fig. 6B). Gene set enrichment analysis (GSEA) confirmed that, compared with the PBS group, the pro-neurogenic gene set was strongly positively enriched in 3D-sEVs-treated NSCs (NES = 1.599, P < 0.001; Fig. 6C, left). This trend remained even when directly comparing 3D-sEVs with 2D-sEVs (NES = 1.413, P = 0.039; Fig. 6C, right). To identify key mediators, we intersected the upregulated DEGs in the 3D-sEVs group with neurogenesis-related gene sets from GeneCards, yielding a set of candidate genes, including NDRG2, FNDC5, ID4, AQP4, and MGLL (Fig. 6D). NDRG2 is primarily characterized as an astrocyte-related regulator that drives glial scar expansion [25], [26], is associated with blood-brain barrier disruption and reactive glial stress, and is not implicated in directed neuronal differentiation [27], [28]. FNDC5 can be cleaved to produce irisin [29], which exerts beneficial metabolic effects by acting on peripheral or central targets [30], [31], [32], [33]. FNDC5 also plays a positive role in neuronal morphological maturation, directed axonal growth, and synaptic plasticity [31], [34].Given the well-established functional advantages of FNDC5 in neural repair, we examined the protein expression levels of FNDC5 and NDRG2 in NSCs following 3D-sEVs intervention by WB. WB analysis revealed that the protein levels of NDRG2 and FNDC5 in NSCs were significantly upregulated in the 3D-sEVs group relative to both the control and 2D-sEVs groups (Fig. 6F). Notably, 3D-sEVs treatment induced a more pronounced upregulation of FNDC5 protein expression in NSCs compared to controls (Fig. 6F). This finding was corroborated by RT-qPCR, which showed that FNDC5 mRNA levels were also significantly elevated (Fig. 6G, P < 0.001). Immunofluorescence staining further confirmed that 3D-sEVs significantly enhanced FNDC5 fluorescence intensity within NSCs. Quantitative analysis revealed that the proportion of SOX-2^+^/FNDC5^+^ cells in the 3D-sEVs group was markedly increased (66.4% ± 2.25%), significantly outperforming the 2D-sEVs group (57.0% ± 2.66%) (Fig. 6H). These results collectively suggest that the therapeutic effects of 3D-sEVs on TBI repair are closely associated with the activation of FNDC5 signaling in NSCs, a mechanistic link that warrants further functional validation.

**Fig. 6:**
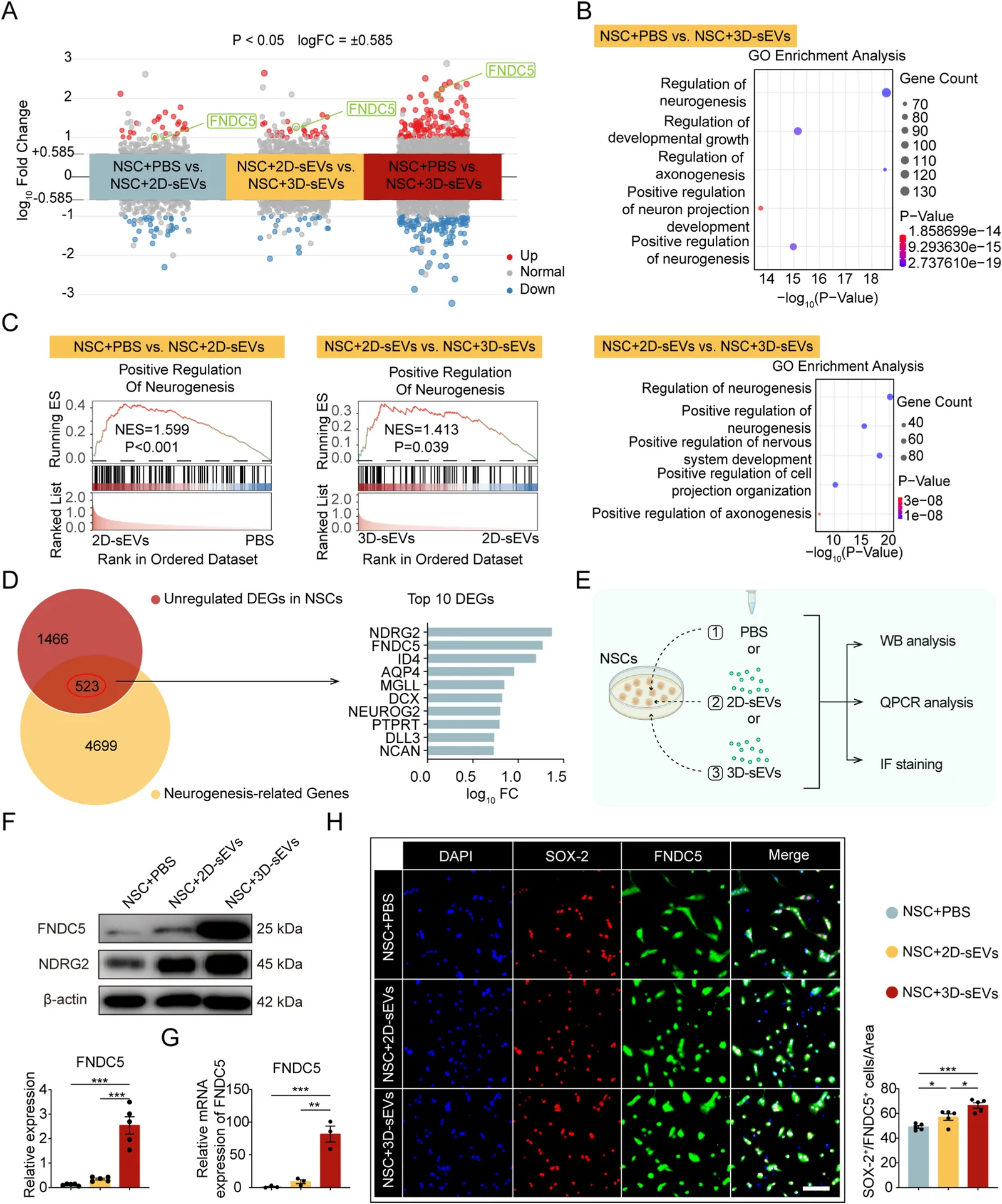
Transcriptomic profiling identifies FNDC5 as the primary downstream target mediating 3D-sEV-induced neurogenesis. (A) Volcano plots of DEGs across comparative NSC groups (red: upregulated; blue: downregulated; green: FNDC5). (B, C) GO enrichment dot plots (B) and GSEA profiles (C) highlighting neurogenesis-related pathways enriched in sEV-treated groups. (D) Venn diagram displaying the intersection of upregulated DEGs with neurogenesis-related genes (left) and ranking of the top 10 candidates by fold change (right). (E) Schematic of the *in vitro* validation workflow. (F, G) Representative Western blot (F), densitometric quantification, and RT-qPCR (G) of candidate targets FNDC5 and NDRG2 in NSCs. (H) Confocal IF images and quantification showing FNDC5 expression (green) in SOX-2^+^ progenitor cells (red). Data are mean ± SD (*p < 0.05, **p < 0.01, ***p < 0.001), scale bar: 100 μm.

### 3.6 Proteomic analysis reveals CREB1 May Be a critical upstream regulator of the FNDC5/PGC-1α axis in 3D-sEVs

To identify candidate effectors within 3D-sEVs that may regulate the PGC-1α/FNDC5 axis, we performed comparative proteomic analysis of 2D-sEVs and 3D-sEVs. Venn analysis showed that 453 proteins were shared by the 3D-sEV proteome and the Vesiclepedia database but were not included in the corresponding 2D-sEV/Vesiclepedia intersection (Fig. 7A). These proteins were therefore selected as 3D-sEV-enriched, Vesiclepedia-annotated candidates.

**Fig. 7:**
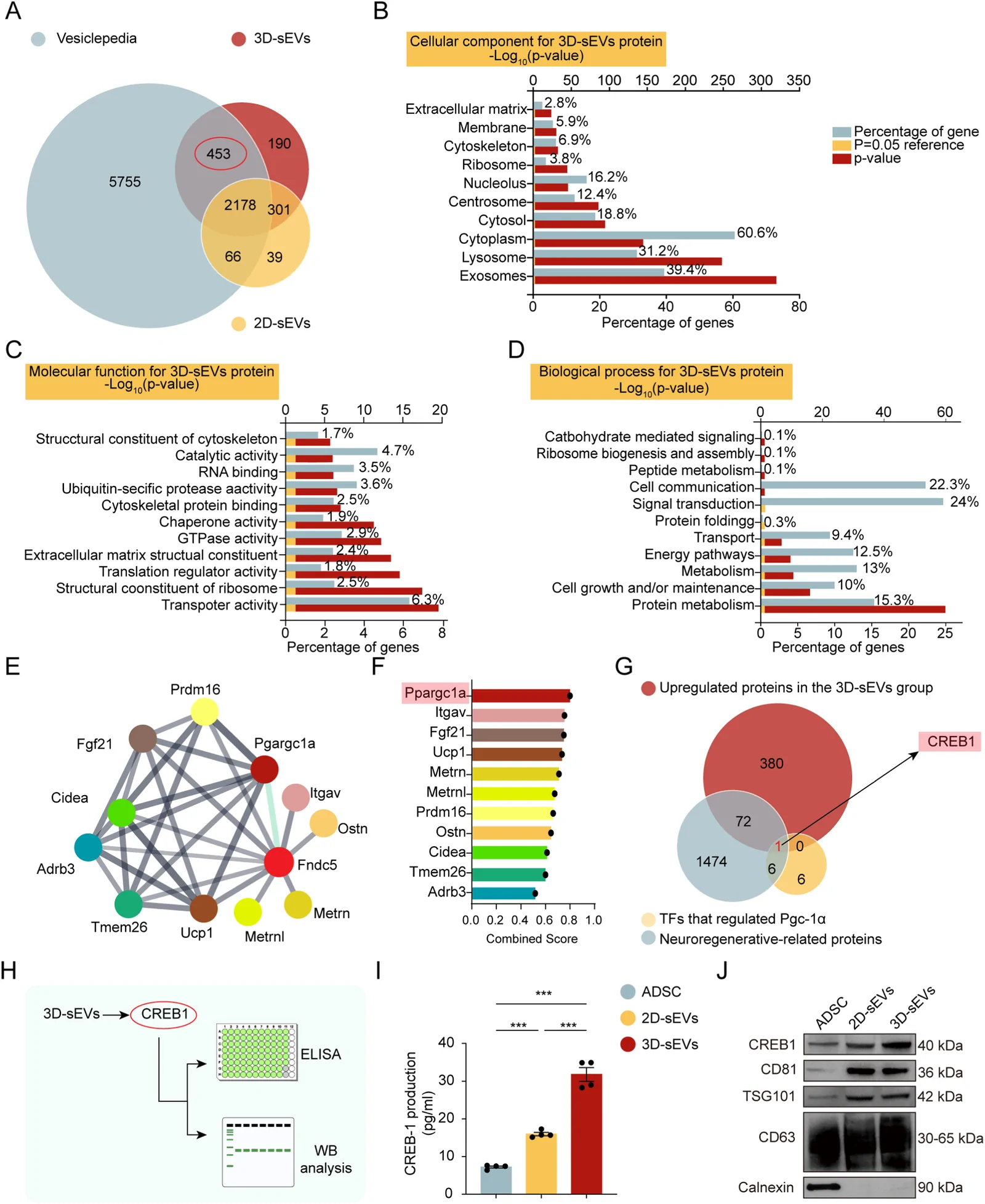
Proteomic characterization of 3D-sEVs identifies CREB1 as the upstream regulator of the PGC-1α/FNDC5 axis. (A) Venn diagram showing protein overlaps between 2D- and 3D-sEVs cross-referenced with Vesiclepedia. (B-D) GO enrichment analysis of 3D-sEV cargo across Cellular Component (B), Molecular Function (C), and Biological Process (D). (E, F) PPI network mapping the FNDC5 interactome (E) and ranking candidates by interaction score (F). (G) Venn diagram intersecting the 3D-sEV-upregulated proteins, PGC-1α transcription factors, and neuroregeneration-related proteins, isolating CREB1. (H) Schematic of CREB1 validation workflow. (I, J) CREB1 quantification via ELISA (I) and Western blot (J) with EV positive markers (CD81, TSG101, CD63) and negative marker (Calnexin). Data are mean ± SD (***p < 0.001).

Gene Ontology analysis showed that these candidates were mainly annotated to the cytoplasm, exosomes, lysosomes, cytoskeleton, and related cellular components (Fig. 7B). At the molecular-function level, transporter activity, catalytic activity, RNA binding, cytoskeletal protein binding, and structural constituent of the ribosome were among the most represented categories (Fig. 7C). At the biological-process level, the candidates were primarily associated with signal transduction, cell communication, energy pathways, metabolism, and protein metabolism (Fig. 7D). These annotations were used to characterize the 3D-sEV cargo profile and were not considered independent evidence of vesicle identity.

To investigate the relationship between FNDC5 and its potential upstream regulators, we constructed an FNDC5-centered protein–protein interaction network using the STRING database. PGC-1α, encoded by Ppargc1a, showed the highest-confidence association with FNDC5 and was therefore selected as the key upstream regulatory node of FNDC5 (Fig. 7E). The top-ranked PGC-1α-associated proteins included Ppargc1a, Itgav, Fgf21, Ucp1, Metrn, Metrnl, Prdm16, Ostn, Cidea, Tmem26, and Adrb3 (Fig. 7F).

We next performed a three-way intersection analysis using the 3D-sEV-upregulated protein set, transcription factors reported to regulate Ppargc1a, and neuroregeneration-related proteins (Fig. 7G). The 3D-sEV-upregulated dataset contained 453 proteins, while the Ppargc1a-regulating transcription-factor dataset contained 13 proteins. The intersection of all three datasets yielded a single protein, CREB1, which was therefore prioritized as the candidate upstream regulator of the PGC-1α/FNDC5 axis.

CREB1 enrichment in sEVs was subsequently validated experimentally. ELISA showed that CREB1 protein production was significantly higher in 3D-sEVs than in 2D-sEVs (Fig. 7H–I). Western blotting further confirmed CREB1 enrichment in 3D-sEVs, together with the expected sEV markers CD81, TSG101, and CD63 and the absence of the cellular contamination marker Calnexin (Fig. 7J). In addition, the TF perturbation analysis showed significant overlap between the 3D-sEV-upregulated gene set and genes downregulated after Creb1 knockdown in mice, including Ppargc1a and Fndc5 (Fig. S17A–B). These results support the prioritization of CREB1 as a potential upstream regulator of the PGC-1α/FNDC5 pathway.

### 3.7 CREB1 contributes to 3D-sEV-induced neural lineage-associated responses

To functionally validate the CREB1/PGC-1α/FNDC5 axis identified above, we pharmacologically inhibited CREB1 using the specific small-molecule inhibitor 666-15 [35] (Fig. 8A–B). WB demonstrated that 3D-sEVs markedly increased the protein levels of CREB1, PGC-1α, and FNDC5, whereas this upregulation was largely abrogated by 666-15 pretreatment (Fig. 8C–D), a finding corroborated by immunofluorescence (Fig. S18A–C). To determine whether this signaling cascade contributes to the changes in neural lineage-marker expression observed in 3D-sEV-treated NSCs induced by 3D-sEVs, we examined the expression of neural lineage markers via RT-qPCR, WB, and immunofluorescence (Fig. 8A). Quantitative analyses revealed that, compared with 3D-sEVs alone, 666-15 administration significantly reduced the expression of DCX, MAP-2, and Olig-2, while markedly elevating GFAP levels (Fig. 8E–H, Fig. S19). Consistently, immunofluorescence staining further confirmed that 666-15 attenuated the 3D-sEV-induced increases in neuronal- and oligodendroglial-lineage markers of 3D-sEVs, as well as their suppression of glial scar formation (Fig. 8I–K). Taken together, these loss-of-function findings support an important contribution of the CREB1/PGC-1α/FNDC5 pathway to the regulation of neural lineage-marker expression in 3D-sEV-treated NSCs and microenvironmental remodeling, establishing this signaling cascade as the central axis governing 3D-sEV-driven TBI repair.

**Fig. 8:**
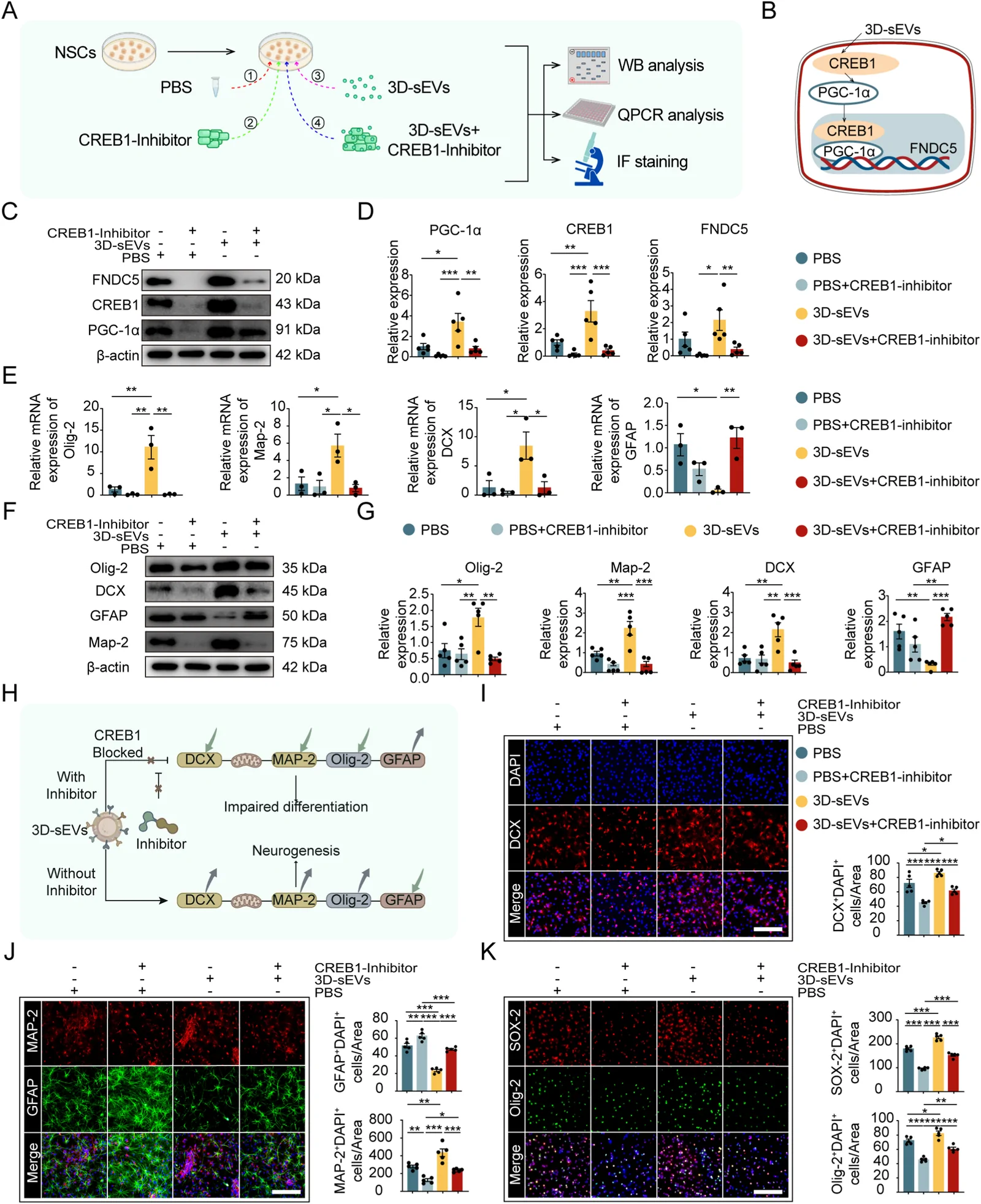
Functional validation of the CREB1/PGC-1α/FNDC5 axis in 3D-sEV-mediated NSC fate regulation. (A, B) Schematics of the *in vitro* loss-of-function experimental design (A) and proposed intracellular signaling model (B). (C, D) Representative Western blot (C) and densitometric quantification (D) of CREB1, PGC-1α, and FNDC5 protein expression under CREB1 inhibition. (E) RT-qPCR analysis of lineage-specific mRNA markers (Olig-2, MAP-2, DCX, and GFAP). (F, G) Representative Western blot (F) and densitometric quantification (G) of neural lineage marker proteins. (H) Schematic summary of cell fate shifts following CREB1 blockade. (I–K) Representative confocal IF images and cell quantifications evaluating immature neuroblasts (DCX^+^, I), neuronal versus astrocytic differentiation (MAP-2^+^ vs. GFAP^+^, J), and oligodendrocyte lineage progression (SOX-2^+^/Olig-2^+^, K). Data are mean ± SD (*p < 0.05, **p < 0.01, ***p < 0.001.) Scale bars: 100 μm.

### 3.8 Donor-cell CREB1 knockdown attenuates the effects of 3D-sEVs on NSC lineage-associated responses

To distinguish the effects of donor-cell CREB1 depletion from the inhibition of endogenous CREB1 in recipient NSCs by 666-15, we silenced CREB1 in ADSCs before 3D bioprinting culture system and sEV isolation (Fig. S20A). CREB1 knockdown in donor ADSCs was confirmed at the protein and mRNA levels (Fig. S20C and E). The resulting 3D-sEVs^sh-CREB1^ retained vesicle-like morphology and expressed TSG101, CD63, and CD9, whereas calnexin was not detected in the sEV preparations (Fig. S20D and F). Compared with 3D-sEVs^sh-NC^, 3D-sEVs^sh-CREB1^ contained less CREB1; PGC-1α and FNDC5 levels in the sEV preparations were also reduced (Fig. S20G).

We next treated NSCs with these sEV preparations (Fig. 9A). Compared with 3D-sEVs^sh-NC^, CREB1-depleted 3D-sEVs reduced the expression of the neuronal markers DCX and MAP-2 and the oligodendroglial marker Olig-2, while increasing the astrocytic marker GFAP, as assessed by Western blotting and immunofluorescence (Fig. 9B–F). These findings indicate that CREB1 knockdown in donor ADSCs attenuates the ability of 3D-sEVs to promote neuronal- and oligodendroglial-lineage-associated responses in NSCs *in vitro*.

**Fig. 9:**
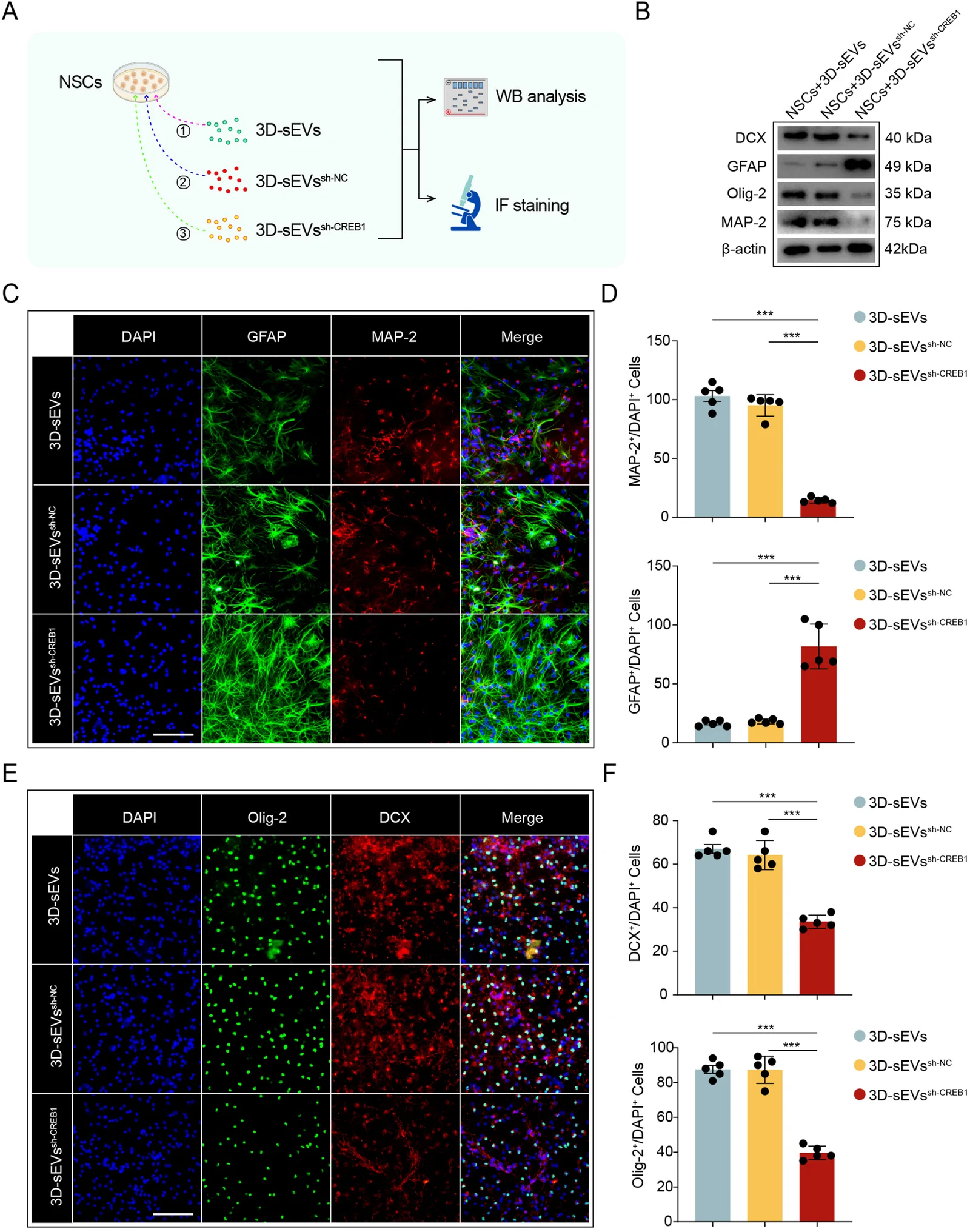
Depletion of CREB1 attenuates 3D-sEV-induced neuronal and oligodendroglial differentiation of NSCs. (A) Experimental scheme depicting the treatment of NSCs with 3D-sEVs, 3D-sEVssh-NC, or 3D-sEVssh-CREB1 for downstream WB and immunofluorescence (IF) analyses. (B) Western blot analysis of immature neuronal (DCX), mature neuronal (MAP-2), astrocytic (GFAP), and oligodendroglial (Olig-2) markers in NSCs following indicated treatments. (C) Representative IF co-staining images of GFAP (green) and MAP-2 (red) in treated NSCs. Nuclei were counterstained with DAPI (blue). Scale bar = 100 μm. (D) Quantitative analysis showing the percentage of MAP-2^+^/DAPI^+^ and GFAP^+^/DAPI^+^ cells across treatment groups. (E) Representative IF co-staining images of Olig-2 (green) and DCX (red) in treated NSCs. Nuclei were counterstained with DAPI (blue). Scale bar = 100 μm. (F) Quantitative analysis showing the percentage of DCX^+^/DAPI^+^ and Olig-2^+^/DAPI^+^ cells across treatment groups. Data are presented as mean ± SEM (n = 5 fields/group). ***P < 0.001.

### 3.9 Pharmacological inhibition of PGC-1α attenuates 3D-sEV-associated changes in NSC lineage markers

To further examine the contribution of PGC-1α activity to the effects of 3D-sEVs on NSCs, we treated NSCs with 3D-sEVs in the presence or absence of the PGC-1α inhibitor SR-18292, with PBS and PBS plus SR-18292 as controls (Fig. S21A). Compared with 3D-sEV treatment alone, co-treatment with SR-18292 reduced the proportions of MAP-2-, DCX-, and Olig-2-positive cells and increased the proportion of GFAP-positive cells (Fig. S21A–D). Western blot analysis showed reduced PGC-1α and FNDC5 protein levels in the 3D-sEV plus SR-18292 group, while CREB1 remained elevated relative to the PBS group (Fig. S21E). These findings support a contribution of PGC-1α activity to the 3D-sEV-associated changes in NSC lineage-marker expression. The accompanying decrease in FNDC5 is consistent with its involvement in the proposed pathway, but does not establish that FNDC5 is functionally required.

## 4 Discussion

In this study, we established an engineered platform that combines coaxial alginate-microfiber culture of ADSCs with GelMA-based local delivery of sEVs. Although the sEVs generated in this system exhibited higher yield, distinct cargo characteristics, and enhanced neuroregenerative activity compared with sEVs obtained from conventional monolayer culture, this comparison should be interpreted as a comparison between two integrated culture platforms rather than as a direct test of dimensionality alone. The 3D group differed from the 2D group in several interrelated features, including cell confinement within alginate microfibers [36], [37], altered cell–cell and cell–matrix interactions [38], changes in oxygen and nutrient diffusion, matrix-associated mechanical cues, and possible shear stimulation during the extrusion process. Such changes in the cellular microenvironment may modify stem-cell properties and secretory profiles [39]. Consistent with this concept, previous studies have reported that specific 3D bioprinting culture system configurations can increase the production and functional activity of stem-cell-derived extracellular vesicles [40], [41]; however, these findings do not necessarily indicate that three-dimensionality itself is the sole determinant of vesicle function. At the mechanistic level, changes in cytoskeletal organization and mechanical signaling under 3D conditions may influence nuclear architecture and YAP-related transcriptional activity, thereby affecting the paracrine behavior of MSCs [42]. In addition, the spheroid-like cellular organization formed within the microfiber environment may alter local oxygenation, nutrient and waste transport, and cadherin-mediated cell–cell contacts, all of which could contribute to changes in ADSC secretion [43]. The transient laminar shear forces generated during microfiber fabrication may also influence cell-cycle status, apoptosis resistance, and the maintenance of stem-cell characters [44], potentially supporting the sustained production of sEVs with relatively consistent properties [45]. Thus, the enhanced cargo profile and therapeutic efficacy observed in the present study most likely reflect the combined effects of the alginate-microfiber architecture, cell organization, matrix-related signals, transport conditions, and bioprinting-associated mechanical stimulation. The current data therefore demonstrate the advantages of the specific coaxial alginate-microfiber culture system used here, but do not establish that the 3D dimension alone is responsible for the observed biological effects. Future factorial experiments incorporating uncoupled controls will be required to determine the individual contributions of dimensionality, alginate encapsulation, cell aggregation, and processing-related mechanical cues.

In the complex and hostile microenvironment of TBI, blood–brain barrier disruption [46] and increased proteolytic activity may accelerate the loss of locally administered biological agents [2], [47], [48]. Directly administered sEVs may undergo rapid dispersion and clearance from the lesion site, limiting the duration of local exposure [12], [49]. GelMA-based hydrogels have consequently been investigated as local depots for encapsulated biological cargo and sustained delivery [50]. Prolonged local exposure to therapeutic sEV preparations may provide continued support for neural repair during the subacute and chronic stages of TBI [51]. Our previous study using the same GelMA formulation showed sustained release and prolonged local DiR-associated fluorescence *in vivo* [4]. In the present study, 2D-sEVs@Gel and 3D-sEVs@Gel exhibited similar release profiles, with approximately 80% of the initially loaded sEV-associated protein recovered in the release medium by day 16. No statistically detectable formulation effect or formulation-by-time interaction was observed, suggesting that sEV origin did not measurably affect the overall GelMA-mediated release profile under the conditions examined. By reducing rapid dispersal from the lesion site, GelMA-mediated delivery may prolong local exposure and potentially reduce the need for repeated intraparenchymal administration. The hydrogel may also provide a temporary three-dimensional matrix within the lesion environment. Nevertheless, persistence of DiR-associated fluorescence should not be interpreted as direct evidence that intact and biologically active sEVs remained present throughout the entire observation period. Moreover, because release was quantified using the BCA assay, the resulting profiles represent sEV-associated protein release rather than the release of intact vesicles. Similarly, the approximately 20% of initially loaded sEV-associated protein that was not recovered in the release medium by day 16 cannot be assumed to represent intact sEVs remaining within the hydrogel. Because GelMA degradation depends on its formulation, crosslinking conditions, and enzymatic environment [16], direct physicochemical characterization remains necessary. The swelling ratio, degradation behavior, and mechanical properties were not directly compared among blank GelMA, 2D-sEVs@Gel, and 3D-sEVs@Gel in the present study, and this should be addressed in future studies.

Several studies have shown that specific 3D bioprinting culture system configurations can significantly boost sEV production by inducing cortical actin depolymerization, thereby removing physical barriers to secretion [52]. Moreover, 3D bioprinting culture system conditions not only enhance sEV yield but also improve their bioactivity [36]. In the context of TBI [53], sEVs derived from mesenchymal stem cells (MSCs) and NSCs have been demonstrated to alleviate neuroinflammation and promote neurogenesis and axonal sprouting [54], [55], [56]. Our previous studies have confirmed that 3D-sEVs, during the acute phase of TBI, can significantly mitigate the cascading inflammatory response triggered by primary injury by suppressing the pro-inflammatory cytokine storm and excessive microglial activation [4], [23]. However, TBI is a continuous pathological process [57]. Even though early neuroprotective strategies effectively slow the spread of secondary injury, extensive clinical observations indicate that patients with TBI commonly still face cognitive dysfunction and impaired motor coordination after entering the subacute and chronic phases. Therefore, the subacute and chronic phases (14 days and beyond) represent the critical stages that determine patients' long-term cognitive and motor outcomes [58]. The reasons for cognitive dysfunction and impaired motor coordination lie in the restricted survival of endogenous NSCs within the injured microenvironment and the aberrant shift of their differentiation trajectories [59], [60], [61]. Studies have shown that after TBI, endogenous NSCs often deviate from the normal neurogenic program [62] and instead differentiate toward astrocytes, ultimately forming dense glial scars that physically impede axonal regeneration. Accordingly, the strategic focus of this study is no longer limited to passive tissue protection but has shifted toward actively activating endogenous neural regeneration.

Neural regeneration is a complex biological process precisely regulated by multi-level signals. Integrating our transcriptomic and proteomic cross-validation, we found that treatment with sEVs generated by the present platform was associated with activation of a transcriptional cascade centered on CREB1 as the core hub and the PGC-1α/FNDC5 axis as the effector arm.

Emerging evidence strongly correlates the suppression of these specific molecules with the exacerbation of TBI severity. Specifically, the phosphorylation level of CREB is inversely proportional to the extent of secondary brain injury [63]. TBI pathologically suppresses CREB activity (such as via the CB1-CREB axis), and more profound CREB inhibition directly exacerbates glutamate excitotoxicity, leading to worse clinical phenotypes [64]. Concurrently, TBI triggers a precipitous decline in PGC-1α expression [65]. Given its critical role as a negative regulator of neuroinflammation, targeted restoration of PGC-1α activity can effectively suppress inflammatory cascades, reduce lesion volume, and alleviate severe motor deficits [65], [66]. Furthermore, TBI triggers a marked downregulation of endogenous FNDC5, whereas its exogenous supplementation or overexpression can effectively rescue damaged neurons through the SIRT3-dependent mitochondrial quality control pathway [67]. Accordingly, our finding that 3D-sEVs deliver high-abundance CREB1 protein directly into NSCs is of profound significance. The CREB1 acts as an initiating signal to transactivate PGC-1α, a master regulator of cellular metabolism [68], [69], [70]. It is well established that the transcriptional coactivator PGC-1α and its upstream regulator CREB1 constitute a classical signaling axis governing mitochondrial homeostasis and energy metabolism [71], and further regulate the expression of the key neuroprotective factor FNDC5 [72]. Moreover, studies have shown that Fndc5 expression in neurons is regulated by PGC-1α, and Pgc1a knockout mice exhibit reduced FNDC5 expression levels in the brain [30], [73]. Our results showed that increased FNDC5 expression was associated with higher levels of DCX, MAP-2, and Olig-2, consistent with enhanced neuronal- and oligodendroglial-lineage-associated responses. Oligodendrocyte precursor cells (OPCs) are known to promote myelin plasticity and repair in the adult brain [74], [75], [76]. Furthermore, elevated FNDC5 expression suppresses GFAP^+^ glial scar hyperplasia. Although glial scar formation during the acute phase can limit the spread of primary injury [77], during the subacute and chronic phases it severely impedes the extension of newborn axons and the reconstruction of functional synaptic networks [78]. These findings suggest that FNDC5 not only inhibits oxidative stress [57] and neuronal apoptosis [67], [79] but also provides the driving force for long-distance axonal extension, dendritic spine remodeling, and the restoration of synaptic plasticity. Studies have demonstrated that adenoviral vector-mediated delivery of FNDC5 to the mouse liver, which elevates circulating irisin levels, induces the expression of Bdnf and other neuroprotective genes in the hippocampus [73]. Although NDRG2 has been reported in limited neuroprotective contexts [80], our Western blot analysis revealed that FNDC5 is more prominently elevated than NDRG2 in NSCs incubated with 3D-sEVs, and FNDC5 exhibits a highly synchronized upregulation trend with PGC-1α following 3D-sEVs intervention. Collectively, these results strongly indicate that the pro-neuroregenerative activity of sEVs generated by this platform is at least partly mediated by the activation of CREB1 and its downstream pathways. The exogenous CREB1 protein delivered into cells by the vesicle preparation used in this study effectively modulates the TBI-associated imbalance in neuronal- and glial-lineage marker expression by specifically activating the PGC-1α/FNDC5 signaling cascade, ultimately reinitiating and driving endogenous neurogenesis.

## 5 Conclusion

In summary, we developed an engineered *in situ* delivery platform that integrates sEVs generated by a coaxial alginate-microfiber culture system with a GelMA hydrogel depot. In the present experimental setting, this platform-derived sEV preparation exhibited increased production and distinct cargo characteristics compared with sEVs obtained from monolayer-cultured ADSCs, consistent with previous observations that engineered 3D bioprinting culture system can modulate stem-cell secretory behavior and extracellular-vesicle function. When incorporated into GelMA, the sEVs showed prolonged local persistence at the injured brain site, which is relevant to the rapid clearance and short biological half-life of free sEVs. The integrated 3D-sEVs@GelMA system increased the numbers of proliferating cells expressing neural stem/progenitor-, neuronal-, and oligodendroglial-lineage-associated markers, reduced astrocytic or glial-scar-associated responses, and improved sensorimotor and cognitive outcomes after TBI.

Mechanistically, our data support a role for CREB1-associated activation of the PGC-1α/FNDC5 signaling axis in the neuroregenerative effects of the sEV preparation used in this study. Pharmacological inhibition of CREB1 substantially attenuated changes in neural lineage-marker expression, indicating that this pathway contributes to, but may not fully account for, the therapeutic activity of the vesicles. Importantly, because the 2D and 3D groups differed not only in spatial dimensionality but also in alginate microfiber encapsulation, cell–cell organization, cell–matrix interactions, transport conditions, and bioprinting-related mechanical cues, the present study demonstrates the benefit of this specific integrated culture and delivery platform rather than establishing that three-dimensionality alone is causative. Similarly, the longitudinal fluorescence data support prolonged persistence of the labeled sEV-associated signal but should not be interpreted as definitive proof of continuous release of intact vesicles or as evidence that GelMA is superior to free 3D-sEV administration in the absence of a corresponding *in vivo* bolus-control group. Future studies using factorial control designs and direct particle-level release measurements will be important for separating the contributions of the individual platform components. Within these limitations, the 3D-sEVs@GelMA system represents a promising cell-free strategy for supporting long-term neural repair after TBI.

## CRediT authorship contribution statement

**Ying Ai:** Writing – original draft. **Qian Zhang:** Writing – review & editing, Data curation, Conceptualization. **Tiange Chen:** Writing – review & editing. **Lin Lin:** Methodology. **Ziyang Chen:** Conceptualization. **Ganzhi Liu:** Investigation. **Jiacheng Liu:** Formal analysis. **Zhuojing Dong:** Data curation. **Yanpeng Jiao:** Data curation. **Jingwei Zhang:** Data curation. **Yi Zhang:** Investigation. **Boxun Liu:** Methodology. **Tao Xu:** Investigation. **Xin Chen:** Methodology. **Jinfang Liu:** Writing – review & editing, Supervision, Resources, Funding acquisition. **Liyang Zhang:** Writing – review & editing, Supervision, Methodology, Resources. **Yuguo Xia:** Writing – review & editing, Supervision, Resources, Formal analysis.

## Ethics approval and consent to participate

All animal experiments were conducted according to the standard operating procedure and complied with the rules of the Animal Care and Use Committees of the Laboratory Animal Research Center at Xiangya Medical School of Central South University, Hunan, China (No. 202110093).

## Funding

This study was kindly funded by the 10.13039/501100001809National Natural Science Foundation of China (82571587, 82201543, XYG; 82371407, LJF; 82674181, ZLY), National Postdoctoral Program for Innovative Talent (BX20220356, XYG), 10.13039/501100002858China Postdoctoral Science Foundation (2022M723562, XYG), 10.13039/501100004735Natural Science Foundation of Hunan Province (2024JJ6657, ZQ), 10.13039/501100004735Natural Science Foundation of Hunan Province (2026JJ40073, XYG), 10.13039/501100004791Shenzhen Science and Technology Program (Grant No. KJZD20230923114302006).

## Declaration of competing interest

The authors declare the following personal relationships which may be considered as potential competing interests: Yi Zhang and Boxun Liu are currently employed by Huamei Biotech Co. Ltd., Shenzhen.
