# Supplementary material for: Sustained delivery of 3D-bioprinted stem cell-derived small extracellular vesicles promotes neural regeneration following traumatic brain injury via the CREB1/PGC-1α/FNDC5 axis
Source: Bioact Mater. 2026 Oct 31;70:44–63. doi: 10.1016/j.bioactmat.2026.10.030 (PMC13743902; doi:10.1016/j.bioactmat.2026.10.030)
Supplement: Supplementary file 1 [file mmc1.doc]

**Sustained Delivery of 3D-Bioprinted Stem Cell-Derived Small Extracellular Vesicles Promotes Neural Regeneration Following Traumatic Brain Injury via the CREB1/PGC-1α/FNDC5 Axis**

**SUPPLEMENTAL MATERIAL**

**1.Materials and methods**

**1.1 Collection and culture of ADSCs**

Adipose tissues required for this study were obtained from the Department of Plastic Surgery at the author's affiliated hospital. Prior to sampling, strict adherence was maintained to the guidelines of the Medical Ethics Committee (Ethics Approval No. 202110093), and informed consent was obtained from the patients or their legal guardians after full disclosure. A 20 mL aliquot of adipose tissue was washed thoroughly with PBS containing antibiotics, centrifuged at 300 × g for 5 minutes, and digested with 0.2% collagenase II (1:3, v/v; Sigma-Aldrich) at 37 °C for 30 minutes with gentle agitation. The enzymatic digestion was terminated by adding complete culture medium containing 10% FBS. The resulting cell suspension was passed through a 100 µm cell strainer and centrifuged. The cell pellet was resuspended in 1 mL of Red Blood Cell lysis buffer for 5 minutes at room temperature, followed by centrifugation at 400 × g for 5 minutes.

The isolated cells were seeded into T75 flasks at a density of 2 × 10⁶ cells/flask and cultured in DMEM/F12 supplemented with 10% FBS (A5256701, Gibco) and 1% penicillin-streptomycin (15140122, Gibco) and 10ug EGF (AF-100-15-100ug, PeproTech) and 10ug (100-18B-100ug, PeproTech) at 37 °C in a humidified atmosphere with 5% CO₂. After 24 hours of incubation, non-adherent cells were discarded, and fresh medium was added to the flasks. The culture medium was replaced every 2-3 days. Upon reaching 70%-80% confluency, the cells were detached using 0.125% trypsin (25200056, Gibco) and passaged at a ratio of 1:2. Cells at passages 3-5 were utilized for all subsequent experiments.

**1.2 Biocompatibility Evaluation of GelMA Hydrogel**

Fixed heart, liver, and kidney tissues were dehydrated through graded ethanol, cleared in xylene, and embedded in paraffin. The embedded tissues were sectioned into 4–5 μm thick slices and mounted onto glass slides. Following deparaffinization and rehydration, the sections were subjected to hematoxylin and eosin (H&E) staining. Briefly, the sections were stained with hematoxylin for nuclear visualization, differentiated in acid‑ethanol, and blued in alkaline solution, followed by counterstaining with eosin for cytoplasmic labeling. The stained sections were then dehydrated, cleared, and mounted with coverslips for microscopic examination of tissue morphology. Strict control of the duration for each step was maintained throughout the entire procedure, and normal tissue sections were included as controls to ensure staining quality.

**1.3 Assessment of NSC Proliferation**

To evaluate the proliferative capacity of NSCs *in vitro*, the EdU Cell Proliferation Imaging Kit (C10337, Thermo Fisher Scientific, Waltham, MA, USA) was utilized according to the manufacturer's protocol. Prior to the assay, NSCs were dissociated into single cells and seeded onto PDL (Sigma, P4832-50ML) coverslips. The cells were incubated with fresh medium supplemented with 10 µM EdU for 2 h at 37 °C in a 5% CO₂ humidified incubator, allowing sufficient incorporation of EdU into the DNA of actively dividing cells. Following incubation, the cells were washed with PBS and fixed with 4% paraformaldehyde. A copper protectant and a fluorescent labeling reaction mixture were sequentially added to catalyze the Click chemistry reaction, covalently conjugating EdU to the fluorescent dye. Subsequently, cell nuclei were counterstained with DAPI for 5 min. Fluorescent images were acquired using an inverted fluorescence microscope (N2-DMi8, Leica, Germany), and the proliferation rate was quantified as the ratio of EdU-positive cells to total DAPI-positive cells using ImageJ software.

To trace cellular proliferation *in vivo*, EdU (E10187, Thermo Fisher Scientific) was administered systemically to the TBI mouse model. Briefly, the EdU powder was dissolved in sterile PBS to prepare a working solution. Mice were administered EdU via intraperitoneal injection at a dosage of 50 mg/kg body weight once daily for 3 consecutive days prior to sacrifice on day 14. Following the final EdU administration, the mice were euthanized and perfused, and 20-µm-thick coronal brain sections were prepared as described in Section 2.7. The brain sections were subjected to the EdU Click chemistry reaction following the kit instructions. To specifically identify proliferating NSCs within the neurogenic niches or lesion site, the EdU staining was coupled with immunofluorescence co-staining for SOX-2 (as detailed in Section 2.14). The sections were analyzed under a confocal microscope to assess the extent of endogenous neural stem cell proliferation.

**1.4 Isolation and culture of NSCs**

Primary NSCs were isolated from the brains of fetal C57BL/6J mice. Pregnant mice at gestational days 13.5–14.5 were euthanized, and the abdominal area was thoroughly disinfected. Under sterile conditions, the uterus was harvested, and the fetuses were isolated. Following decapitation of the fetuses, the fetal brains were rapidly dissected in ice-cold sterile PBS. Fetal mouse brain tissues were collected under a stereomicroscope. The tissue fragments were enzymatically digested using Accutase (AT104, Innovative Cell Technologies) at 37 °C for 15–30 min, accompanied by gentle mechanical pipetting. The enzymatic digestion was gently terminated by dilution with an excess volume of cold sterile PBS or basal medium. The cell suspension was then passed through a 70 µm cell strainer to remove undigested tissue debris and centrifuged at 150 × g for 5 min. Subsequently, the cell pellet was resuspended in NSC culture medium (CM) containing the following components: DMEM/F12 (C11330500, Gibco, Grand Island, USA), 2% B27 (12587010, Gibco), 20 ng/mL EGF (CYT-554, ProSpec), 20 ng/mL FGF (CYT-386, ProSpec), 5 ug/mL heparin (H3149, Sigma-Aldrich, St. Louis, USA), and 1% Pen-Strep (10378016, Thermo Fisher Scientific, Waltham, MA, USA). Finally, the cells were seeded into 25 cm² culture flasks (707013, Nest) at a density of 5×10^4^ cells/mL and maintained in an incubator at 37℃ with 5% CO2. Half of the culture medium was replaced every two days, and the cells were passaged every 5 to 6 days. For subculture, neurospheres at the logarithmic growth phase were collected together with the medium into centrifuge tubes. After centrifugation, the supernatant was discarded, and Accutase (AT104, Innovative Cell Technologies) cell dissociation solution was added with gentle pipetting for digestion. Following digestion, the cell suspension was gently pipetted 15-20 times until no obvious neurosphere particles were visible to the naked eye, and the digestion was then terminated. After another centrifugation to completely remove residual digestion solution, the cell pellet was resuspended in adherent culture medium. The resuspended cells were seeded at a density of 1×10^5^cells/cm2 onto culture dishes pre-coated with PDL(Sigma, P4832-50ML) solution one day in advance.

**1.5 Enzyme-Linked Immunosorbent Assay (ELISA)**

The protein levels of CREB1 in ADSCs, 2D-sEVs, and 3D-sEVs were quantitatively determined using a commercially available Human CREB1 ELISA Kit (A00577-1, Boster Biological Technology, Wuhan, China) strictly according to the manufacturer’s instructions. Briefly, appropriately diluted standard solutions and experimental samples were added to the pre-coated 96-well microplates and incubated at 37°C for 90 min. Following plate washing, a biotinylated anti-human CREB1 detection antibody was added and incubated at 37°C for 60 min. After thoroughly washing the unbound antibodies, the Avidin-Biotin-Peroxidase Complex (ABC) was added for a 30-min incubation at 37°C. Subsequently, the plates were washed again, and the TMB (3,3',5,5'-tetramethylbenzidine) color-developing agent was applied and incubated in the dark to catalyze the enzymatic colorimetric reaction. The reaction was terminated by adding the stop solution. All sample measurements were normalized to the total protein concentration, which was determined prior via the BCA method. Both standard curves and experimental samples were evaluated in triplicate wells to ensure assay reliability. Finally, the optical density (OD) was immediately read at 450 nm using a SpectraMax i3x multimode microplate reader (Molecular Devices, USA).

**1.6 RT-qPCR analysis**

To evaluate the expression of target genes in NSCs, total RNA was extracted using RNAex Pro reagent (AG21101; Accurate Biotechnology, Hunan, China) according to the manufacturer’s protocol. Then reverse transcription reactions were performed using 500 ng RNA and an Evo M-MLV RT Premix kit (Accurate Biotechnology) according to instructions. SYBR Green Pro Taq HS Kit (Accurate Biotechnology) and 0.4 µM of each primer pair were used to amplify the cDNA, which was then evaluated in an ABI 7500 real-time PCR system (Applied Bioscience, Foster City, CA, USA). The results were analyzed using the ΔΔCt method and expressed as the ratio relative to the internal control, β-actin. The specific primer sequences used in this study were synthesized by Sangon Biotech, China and are detailed in Table S1.Data presented are the average of three independent experiments.

**1.7 Western Blot Analysis**

To characterize the isolated sEVs and evaluate the specific enrichment of cargo proteins, Western blotting was performed. The sEVs or ADSC lysates were homogenized in RIPA lysis buffer (P0013C, Beyotime Biotechnology, China) supplemented with a protease inhibitor cocktail (Beyotime). Protein concentrations were determined using a BCA assay kit (P0012S, Beyotime). Equal amounts of protein (20 μg per lane) were separated by 12% SDS-PAGE and transferred onto 0.22-μm PVDF membranes. After blocking with 5% non-fat milk in TBST for 1 h at room temperature, the membranes were incubated overnight at 4℃ with primary antibodies against classical exosomal markers and the targeted effector protein: anti-CD9 (1:1,000; ab236630; Abcam), anti-CD81 (1:1,000; A5270, ABclonal), anti-TSG101 (1:1,000; ab125011, Abcam), anti-CD63 (1:1,000; ab68418, Abcam), anti-FNDC5 (1:1,000; NBP2-14024, Novus Biologicals), and the endoplasmic reticulum-negative control anti-Calnexin (1:1,000; A15631, ABclonal). Following three washes with TBST, the membranes were probed with HRP-conjugated secondary antibodies (1:5,000) for 1 h at room temperature. Protein bands were visualized using a ChemiDoc™ MP Imaging System (Bio-Rad, Hercules, CA, USA) with an enhanced chemiluminescence substrate. To verify the neural differentiation direction and elucidate the underlying CREB1/PGC-1α/FNDC5 signaling cascade in NSCs after different treatments, Western blotting was similarly performed. The primary antibodies included anti-DCX (1:1,000; ab18723, Abcam), anti-MAP-2 (1:1,000; 4542S, CST), anti-Olig-2 (1:1,000; 39588S, CST), anti-GFAP (1:1,000; GB11096-100, Servicebio), anti-CREB1 (1:1,000; ab178322, Abcam), and anti-PGC-1α (1:1,000; ab131390, Abcam). The obtained band intensities were quantified utilizing ImageJ software (NIH, Bethesda, MD, USA) and normalized to β-actin (1:1,000; GB15003-100, Servicebio), which served as the internal loading control. All experiments were independently repeated three times.

**1.8 Immunofluorescence staining**

During the staining procedure, the samples were first permeabilized with Triton X-100 (SOLARBIO, Catalog No. T8200) and then washed three times with PBS. Subsequently, the samples were blocked with 5% bovine serum albumin (BSA, Sigma) and incubated with the following primary antibodies: anti-CREB1 (ab178322, Abcam), anti-PGC-1α (ab131390, Abcam), anti-FNDC5 (NBP2-14024, NOVUS), anti-DCX (ab18723, Abcam), anti-MAP-2 (4542s, Cell Signaling Technology), anti-Olig-2 (39588S, CST), and anti-GFAP (GB11096-100, Servicebio). The samples were then incubated with secondary antibodies (Invitrogen) for 1 hour. Furthermore, to identify the capacity of NSCs to internalize sEVs, the samples, after permeabilization and blocking, were incubated with a primary antibody against SOX-2 (1:200, ab97959, Abcam) overnight at 4°C. On the following day, the samples were incubated with a fluorophore-conjugated secondary antibody (Alexa Fluor 488-conjugated goat anti-rabbit IgG, 1:500, Abcam) for 1 hour at room temperature in the dark. Cell nuclei were counterstained with DAPI (Sigma). Images were captured using a fluorescence microscope (DM6B, Leica, Germany).

**1.9 Transwell assay**

The migratory capacity of NSCs was assessed using a Transwell chamber system, in which the upper and lower compartments were separated by a polycarbonate membrane with 8-µm pores (Corning, NY, USA). The lower chamber was filled with DMEM supplemented with 10% fetal bovine serum. NSCs (2 × 10⁴ cells) pretreated with NSCs+PBS, NSCs+2D-sEVs, or NSCs+3D-sEVs were seeded into the upper chamber in serum-free DMEM. Following a 24‑hour incubation at 37 °C, the cells on the upper membrane surface were fixed with 4% paraformaldehyde for 30 minutes at room temperature and subsequently removed by gentle wiping with a cotton swab. Migrated cells adherent to the lower membrane surface were stained with crystal violet solution (Cat# G1014, Wuhan Servicebio Technology) for 30 minutes at room temperature. For each well, the number of migrated cells was counted in five randomly selected fields under a light microscope at 20× magnification, and the migration rate was subsequently calculated.

**1.10 Evaluation of brain tissue loss**

To evaluate cortical tissue loss, representative coronal brain sections prepared as described in Section 2.7 were utilized. For each mouse, six sections spanning the entire rostrocaudal extent of the lesion core at strictly defined intervals of 20 µm were selected. These sections were subjected to Nissl staining using a cresyl violet acetate solution (C5042, Sigma-Aldrich, St. Louis, MO, USA) according to the manufacturer's instructions.To accurately quantify tissue loss while eliminating the confounding effects of post-traumatic brain edema, an indirect volume calculation method based on Cavalieri's principle was employed. Stained sections were imaged, and the respective areas were manually delineated using ImageJ software (version 1.53r). For each section, the intact tissue area of the uninjured contralateral hemisphere (left side) and the remaining intact tissue area of the injured ipsilateral hemisphere (right side) were measured.The total volume of each hemisphere was calculated by integrating the respective areas across the six sections and multiplying by the distance between adjacent sections. The absolute tissue loss volume was defined as the volume of the contralateral hemisphere (V_c_) minus the preserved volume of the ipsilateral hemisphere (V_i_). Finally, the extent of brain injury was expressed as the percentage of tissue loss relative to the contralateral volume: Tissue Loss (%) = [(V_c_ - V_i_) / V_c_] × 100.

**1.11 Behavioral Evaluation**

Motor coordination was assessed via the rotarod test and cognitive performance and spatial memory were evaluated using the Morris Water Maze (MWM). Mice underwent rotarod test and Morris water maze test for up to 28 days post-TBI to evaluate motor function as well as cognitive and memory function. Before the CCI procedure, mice were trained for 3 days to establish a baseline standard. For the rotarod test, an automated rotarod apparatus (XR-6C Mouse Rotarod Fatigue Apparatus, Shanghai Xinruan) was used in an accelerated speed mode (rotational speed increasing uniformly from 4 rpm to 40 rpm over 5 minutes). The test was performed on days 1, 3, 7, 14, 21, and 28 post-surgery, and the latency to fall from the rotarod was recorded. At each time point, each mouse underwent three repeated trials (with an interval of 15–20 minutes), and the average latency to fall from the three trials was taken as the motor function score for that mouse on that day. The Morris water maze test consisted of a place navigation test and a spatial probe test, conducted using the Morris water maze apparatus (XR-XM101, Shanghai Xinruan Information Technology Co., Ltd.). The place navigation test was performed on days 24–28 post-TBI for 5 consecutive days. At a fixed time each day, starting randomly from four different designated entry points facing the pool wall, each mouse was gently placed into the water from the same entry point outside the third quadrant, with the head facing the pool wall. The swimming trajectory, escape latency, and swimming path characteristics were recorded. On day 28, the spatial probe test was conducted by removing the platform and releasing the mouse from the same entry point used during the training phase, allowing the mouse to swim freely in the pool for 60 seconds. Video tracking was used to record and analyze parameters including the duration of time spent in the target quadrant, the number of crossings over the original platform position, and the average swimming speed.

**1.12 Transcriptomic Sequencing and Analysis**

Total RNA quality was assessed using a NanoPhotometer, Qubit 2.0 fluorometer, and Agilent 2100 Bioanalyzer. Polyadenylated mRNA was enriched using oligo(dT), fragmented to approximately 200 bp, and used to construct libraries with the Illumina NEBNext® Ultra™ RNA Library Prep Kit. Libraries were sequenced on an Illumina platform using paired-end 150-bp reads. Clean reads were aligned to the mouse reference genome GRCm38 (mm10) using HISAT2, followed by transcript assembly with StringTie. Gene-level raw counts were generated using featureCounts; FPKM values were calculated separately for expression visualization. DESeq2 was used to identify DEGs, with Padj < 0.05 and |log10FC| > 1 as the significance criteria. GO enrichment and GSEA were performed using clusterProfiler with org.Mm.eg.db, and msigdbr with fgsea, respectively.

**1.13 Proteomic Analysis of ADSC-sEVs**

To characterize and compare the protein cargo of 2D-sEVs and 3D-sEVs, label-free quantitative proteomic analysis was performed by Wayen Biotechnologies (Shanghai) Co., Ltd. ADSC-derived sEVs were isolated by ultracentrifugation, and sufficient quantities of the purified sEV samples were submitted for analysis. Vesicular proteins were extracted from each sample and processed for mass spectrometric analysis. Briefly, the extracted proteins were reduced and alkylated, followed by tryptic digestion to generate peptides. The resulting peptide mixtures were purified and separated by high-performance liquid chromatography (HPLC), and the separated peptides were analyzed by liquid chromatography–tandem mass spectrometry (LC–MS/MS). The acquired raw mass spectrometry data were searched against the relevant *Homo sapiens* UniProt database for protein identification. Relative protein abundance was determined using label-free quantification, enabling comparison of the protein profiles of 2D-sEVs and 3D-sEVs. Further analyses of the identified proteins are described in the corresponding sections of the manuscript.

2. **Results**

**Figure S1. *In vitro* cytocompatibility and *in vivo* biosafety of GelMA hydrogel.** (A, B) Representative Live/Dead staining images of (A) HT22 cells and (B) BV2 cells after 1 and 7 days of culture (green: live cells; red: dead cells). Scale bars = 50 µm. (C) Representative H&E-stained histological sections of major organs (heart, lung, liver, spleen, and kidney) from the Sham, TBI, and TBI+GelMA groups. Scale bar = 100 µm. (D) Quantitative analysis of serum biochemical markers for liver (ALT, AST) and kidney (BUN, CREA) functions (n = 5). Data are presented as mean ± SD. ns: not significant.

**Figure S2. In vivo degradation kinetics of GelMA hydrogel and sustained retention of sEVs.** (A) Gross morphological observation of GelMA hydrogel degradation and brain tissues at indicated time points (Days 1, 3, 7, 14, and 28 post-TBI). (B) Quantitative curve showing the in vivo degradation profile of the hydrogel over 28 days. (C) Representative in vivo fluorescence images (IVIS) tracking the spatial-temporal retention and release kinetics of fluorescently labeled sEVs at the lesion site for up to 24 days. (D) Quantitative analysis of fluorescence intensity decay over time at the injury site (n = 5). Data are presented as mean ± SD.

**Figure S3. Western blot characterization of ADSC-derived sEVs.** (A) Representative Western blots of CD81, TSG101, CD63, and calnexin in ADSC lysates and sEVs isolated from 2D or 3D ADSC cultures. Calnexin was included as a cellular contamination marker. (B) Corresponding original blot images. Red boxes indicate the regions displayed in A; molecular masses are shown in kilodaltons (kDa).

**Figure S4. In vitro cumulative release profiles of sEVs from hydrogels.** Cumulative release kinetics of sEVs from 2D-sEVs@GelMA and 3D-sEVs@GelMA hydrogels over a period of 16 days. Data are presented as mean ± SEM (n = 3).

**Figure S5. Histopathological and behavioral evaluation at 28 days post-TBI (chronic phase)** (A) Representative coronal brain sections (Nissl staining) and corresponding reconstructed lesion profiles across treatment groups. (B-D) Linear regression analyses correlating rotarod performance (latency to fall and total distance) with IF counts of newly generated neural cells (DCX^+^/EdU^+^, NeuN^+^/EdU^+^, and SOX-2^+^/EdU^+^). (E-G) Linear regression analyses correlating Morris water maze (MWM) performance (learning time and platform crossings) with IF cell counts (DCX^+^/EdU^+^, NeuN^+^/EdU^+^, and SOX-2^+^/EdU^+^). (H) Swimming speed during the MWM learning phase (ns: non-significant).

**Figure S6. Nissl staining at 14 days after CCI.** (A) Representative coronal brain sections and higher-magnification images of the peri-lesional cortex from the Sham+PBS, CCI+PBS, CCI+GelMA, CCI+2D-sEVs@GelMA, and CCI+3D-sEVs@GelMA groups. Scale bar, 50 μm for the enlarged cortical images. (B) Quantification of Nissl-positive cells in the peri-lesional cortex. (C) Representative Nissl-stained images of the hippocampal CA1, dentate gyrus (DG), and CA3 regions. Scale bars, 50 μm. (D) Quantification of Nissl-positive cells in the CA1, DG, and CA3 regions. Data are presented as mean ± SEM (n = 5 animals per group). *P < 0.05, **P < 0.01, ***P < 0.001; ns, not significant.

**Figure S7. Nissl staining at 28 days after CCI.** (A) Representative coronal brain sections and higher-magnification images of the peri-lesional cortex from the Sham+PBS, CCI+PBS, CCI+GelMA, CCI+2D-sEVs@GelMA, and CCI+3D-sEVs@GelMA groups. Scale bar, 50 μm for the enlarged cortical images. (B) Quantification of Nissl-positive cells in the peri-lesional cortex. (C) Representative Nissl-stained images of the hippocampal CA1, dentate gyrus (DG), and CA3 regions. Scale bars, 50 μm. (D) Quantification of Nissl-positive cells in the CA1, DG, and CA3 regions. Data are presented as mean ± SEM (n = 5 animals per group). *P < 0.05, **P < 0.01, ***P < 0.001; ns, not significant.

**Figure S8. 3D-sEVs@Gel attenuates lesion volume and promotes endogenous neurogenesis at the subacute phase (day 14) post-TBI.** (A) Representative serial brain sections (Nissl staining) delineating cortical lesion boundaries (outlined in red) and tissue loss across different treatment groups (Sham+PBS, CCI+PBS, CCI+GelMA, CCI+2D-sEVs@Gel, and CCI+3D-sEVs@Gel) at day 14 post-injury. (B) Quantitative analysis of proliferating neural stem/progenitor cell (DCX^+^/EdU^+^ and SOX-2^+^/EdU^+^) density per unit area around the lesion site at day 14 post-TBI (n = 5). (C, D) Linear regression and correlation analyses between neurogenic markers DCX^+^/EdU^+^ (C) and SOX-2^+^/EdU^+^ (D) and behavioral performance metrics (Time and Distance). *Data are presented as mean ± SEM. *p<0.05, **p<0.01, ***p<0.001.

**Figure S9. 3D-sEVs@Gel enhances NSC proliferation and oligodendroglial differentiation at day 14 post-TBI.** (A) Immunofluorescence and quantification of SOX-2^+^/Olig-2^+^ progenitor cells in perilesional regions at day 14 post-TBI (n = 5). Scale bar = 100 µm. (B) Linear correlations between SOX-2^+^/Olig-2^+^ cell density and behavioral performance (Time and Distance). (C) Immunofluorescence and quantification of proliferating NSCs SOX-2^+^/EdU-2^+^) in neurogenic niches (n = 5). Scale bar = 100 µm. (D) Linear correlations between SOX-2^+^/EdU-2^+^ cell density and behavioral performance. *Data are mean ± SEM. *p<0.05, **p<0.01, ***p<0.001.

**Figure S10. GelMA-Encapsulated 3D-sEVs Promote Neural Regeneration and Cognitive Recovery at 28 Days Post-TBI (Chronic Phase).** (A, D) Immunofluorescence imaging and quantification within the peri-injury cortex show that treatment with GelMA-encapsulated 3D-sEVs significantly increases the densities of (A) SOX-2^+^/Olig-2^+^ (oligodendrocyte progenitor lineage) and (D) newly generated SOX-2^+^/EdU^+^ in neurogenic niches (n = 5). (B, C, E, F) Linear regression analyses demonstrate positive correlations between the density of (B, C) SOX-2^+^/Olig-2^+^ and (E, F) SOX-2^+^/EdU^+^ cells with behavioral performance indices in the Morris water maze (probe trial latency and distance, training trial learning time, and platform crossing frequency). (*p<0.05, **p<0.01, ***p<0.001). Scale bar = 100 µm.

**Figure S11. GAP-43 and MAP-2 immunoreactivities in the CCI lesion area and their associations with motor function at 14 days after CCI.** (A) Representative immunofluorescence images of GAP-43 (green) in the CCI lesion area. Nuclei were counterstained with DAPI (blue). (B) Quantification of GAP-43 fluorescence intensity. (C) Representative immunofluorescence images of MAP-2 (green) in the CCI lesion area. (D) Quantification of MAP-2 fluorescence intensity. (E) Correlations of GAP-43 fluorescence intensity with rotarod latency to fall and distance traveled. (F) Correlations of MAP-2 fluorescence intensity with rotarod latency to fall and distance traveled. Image acquisition and quantitative analyses were performed by an investigator blinded to the group allocation. Each dot represents one animal (n = 5 animals per group), and data are presented as the mean ± SEM. Group comparisons were performed using one-way ANOVA followed by Tukey’s multiple-comparisons test. Correlations were evaluated using Pearson’s correlation analysis; solid and dashed lines indicate the fitted regression line and 95% confidence interval, respectively. Scale bars, 100 μm. *P < 0.05, **P < 0.01, and ***P < 0.001.

**Figure S12. GAP-43 and MAP-2 immunoreactivities in the CCI lesion area and their associations with spatial learning and memory at 28 days after CCI.** (A) Representative immunofluorescence images of GAP-43 (green) in the CCI lesion area. Nuclei were counterstained with DAPI (blue). (B) Quantification of GAP-43 fluorescence intensity. (C) Representative immunofluorescence images of MAP-2 (green) in the CCI lesion area. (D) Quantification of MAP-2 fluorescence intensity. (E) Correlations of GAP-43 fluorescence intensity with escape latency and the number of platform crossings in the Morris water maze. (F) Correlations of MAP-2 fluorescence intensity with escape latency and the number of platform crossings. Image acquisition and quantitative analyses were performed by an investigator blinded to the group allocation. Each dot represents one animal (n = 5 animals per group), and data are presented as the mean ± SEM. Group comparisons were performed using one-way ANOVA followed by Tukey’s multiple-comparisons test. Correlations were evaluated using Pearson’s correlation analysis; solid and dashed lines indicate the fitted regression line and 95% confidence interval, respectively. Scale bars, 100 μm. *P < 0.05, **P < 0.01, and ***P < 0.001.

**Figure S13. 3D-sEVs Enhance the Migratory Capacity of NSCs i*n Vitro*.** (A) Scratch wound healing assay. Representative micrographs show the scratch area (dashed lines indicate the wound edges) in NSCs treated with PBS (control), 2D-sEVs, or 3D-sEVs at 0 h and 24 h. (B) Transwell migration assay. Representative micrographs of migrated cells on the bottom surface of the Transwell membrane. (C) Quantitative analysis. Left panel: migration distance normalized to the control group (percentage); right panel: number of migrated cells. Data are presented as mean ± SD, ***p < 0.001 (one‑way ANOVA). Scale bars are indicated in the figures.

**Figure S14. Transcriptomic profiling and neurogenesis pathway analysis in 3D-sEVs-treated NSCs.** (A) Heatmap of differentially expressed genes (DEGs) between 3D-sEVs-treated and 2D-sEVs-treated NSCs (n = 3 per group). Color scale indicates normalized gene expression levels (Z-score). (B) Gene Set Enrichment Analysis (GSEA) plot showing significant enrichment of the "Neurogenesis" gene set in the 3D-sEVs group (NES = 1.494, ***P<0.001). (C) Relative expression of NDRG2 in NSCs across different treatment groups (PBS, 2D-sEVs, and 3D-sEVs). Data are presented as mean ± SEM (**P < 0.01).

**Figure S15. Transcriptomic profiling of NSCs treated with 2D-sEVs.** (A) Heatmap of differentially expressed genes (DEGs): Hierarchical clustering showing DEG expression profiles between the NSCs+PBS and NSCs+2D-sEVs groups (n = 3 per group). The color scale indicates relative expression levels (red, upregulated; blue, downregulated). (B) Gene Ontology (GO) enrichment analysis: Bubble plot displaying key biological processes and cellular components enriched by DEGs, including regulation of neurogenesis, developmental growth, regeneration, and synapse-related pathways. Dot size corresponds to gene count, and color represents the P-value(log_10_(*P-Value*)).

**Figure S16. Transcriptomic profiling of NSCs treated with 3D-sEVs.** (A) Heatmap of differentially expressed genes (DEGs): Hierarchical clustering showing DEG expression profiles between the NSCs+PBS and NSCs+3D-sEVs groups (n = 3 per group). The color scale represents relative gene expression levels (red, upregulated; blue, downregulated).

**Figure S17. Transcription factor enrichment and target gene analysis of 3D-sEV-mediated regulation.** (A) Transcription factor perturbation enrichment analysis: Scatter plot of enriched transcription factors (TFs). Highlighted are the target TF CREB1 (Adj.*P* = 0.012, red) and key neurogenesis master TFs (e.g., NEUROG2, ASCL1; yellow). The dashed line indicates the significance threshold (Adj.*P* = 0.05). (B) Venn diagram: Identification of overlapping genes between 3D-sEV-upregulated genes (184 total) and CREB1 knockdown (KD)-downregulated genes from dataset GSE17478, revealing 8 co-regulated targets (including FNDC5, AQP4, and HOPX). (C) Volcano plot: Differential gene expression profiling between 3D- and 2D-cultured ADSCs (log_2_FC>1, *P<0.05). Red, yellow, and grey dots represent upregulated, downregulated, and non-significantly changed genes, respectively.

**Figure S18. Inhibition of CREB1 attenuates 3D-sEV-mediated upregulation of neurogenesis and metabolic markers in NSCs.** (A) Expression of SOX-2 and CREB1: Representative immunofluorescence images and quantitative density of SOX-2^+^ (red) and CREB1^+^ (green) co-labeled NSCs treated with PBS, PBS + CREB1 inhibitor, 3D-sEVs, or 3D-sEVs + CREB1 inhibitor. (B) Expression of SOX-2 and PGC-1α: Representative immunofluorescence images and quantitative density of SOX-2^+^ (red) and PGC-1α^+^ (green) co-labeled NSCs across the four treatment groups. (C) Expression of SOX-2 and FNDC5: Representative immunofluorescence images and quantitative density of SOX-2^+^ (red) and FNDC5^+^ (green) co-labeled NSCs across the four treatment groups. Scale bars = 50 μm. Data are presented as mean ± SEM; n = 5 per group. *P < 0.05, **P < 0.01, ***P < 0.001.)

**Figure S19. Uncropped Western blot images corresponding to Figure 5E.** Blots show Olig-2, DCX, MAP-2, GFAP, and β-actin in NSCs treated with PBS, 2D-sEVs, or 3D-sEVs. Red boxes mark the band regions presented in Figure 5E. Molecular weight markers are indicated in kDa; β-actin is shown as the loading control.

**Figure S20. Generation and characterization of CREB1-depleted 3D-sEVs.** (A) Schematic diagram illustrating the workflow for transfecting ADSCs with sh-CREB1, 3D culture, and subsequent isolation of 3D-sEVssh-CREB1 via ultracentrifugation. (B) Representative brightfield and fluorescence images of ADSCs transfected with GFP-tagged lentivirus. Scale bar = 50 μm. (C) Western blot analysis evaluating the knockdown efficiency of different sh-CREB1 sequences in ADSCs. (D) Transmission electron microscopy (TEM) images showing the typical cup-shaped morphology of 3D-sEVssh-NC and 3D-sEVssh-CREB1. Scale bar = 100 nm. (E)qRT−PCR quantification of relative CREB1 mRNA expression levels in ADSCs, ADSCssh-NC, and ADSCssh-CREB1. (F) Western blot analysis of CREB1 cargo, exosomal marker proteins (TSG101, CD63, and CD9), and the cellular negative marker Calnexin indonor cells and their corresponding 3D−sEVs. (G) Western blot analysis showing the protein levels of CREB1 and its downstream targets (FNDC5 and PGC−1α) in NSCs + 3D-sEVs, NSCs + 3D-sEVssh-NC, and NSCs + 3D−sEVssh-CREB1. Data are presented as mean ± SEM (n = 3 per group). P < 0.01.

**Figure S21. SR-18292 attenuates 3D-sEV-associated changes in NSC differentiation markers.** (A) Representative immunofluorescence images of GFAP (green) and MAP-2 (red), with nuclei stained by DAPI (blue). (B) Quantification of MAP-2- and GFAP-positive cells relative to DAPI-positive cells. (C) Representative images of Olig-2 (green) and DCX (red), with DAPI-stained nuclei (blue). (D) Quantification of DCX- and Olig-2-positive cells relative to DAPI-positive cells. (E) Representative Western blots and densitometric analysis of CREB1, PGC-1α, and FNDC5; protein levels were normalized to β-actin. Data are presented as mean ± SEM from 5 independent experiments. Statistical comparisons are indicated by brackets; **P < 0.01, ***P < 0.001. Scale bars: 100 μm.
